# Effectiveness of sports injury prevention programs for adolescent and children football players: a meta-analysis and systematic review

**DOI:** 10.7717/peerj.21319

**Published:** 2026-06-01

**Authors:** HongYu Yang, Xianming Ding, Yue Fan, Xiao Wang, Jia Hui Li

**Affiliations:** 1 School of Football, Chengdu Sport University, Chengdu, Sichuan, China; 2 School of Physical Education, Jishou University, Jishou, Hunan, China

**Keywords:** Injury prevention, Youth athletes, Children, Football, Meta-analysis, Systematic review

## Abstract

**Background:**

Children and adolescent football players are at increased risk of sports-related injuries due to ongoing physical and neuromuscular development. Although various injury prevention programs have been proposed, evidence regarding their effectiveness in these populations remains limited. This systematic review and meta-analysis aimed to evaluate the effectiveness of structured injury prevention programs in reducing overall and site-specific injury incidence among youth football players.

**Methods:**

A systematic search of randomized controlled trials (RCTs) was conducted across Cochrane Library, PubMed, EBSCO, Embase, and Web of Science. Eligible studies included football players aged 3–18 years receiving structured injury prevention interventions. The primary outcome was overall injury incidence, and secondary outcomes included lower-limb, knee, ankle, muscle, hip/groin, contact, and non-contact injuries. Pooled estimates were calculated as incidence rate ratios (IRRs) based on exposure hours.

**Results:**

Nine RCTs involving 16,636 participants and 1,248,381 exposure hours were included. Injury prevention programs significantly reduced overall injury incidence (IRR = 0.62, 95.0% CI [0.49–0.78]; *I*^2^ = 80.0%). Significant reductions were also observed for lower-limb (IRR = 0.68), knee (IRR = 0.67), and ankle injuries (IRR = 0.72). Muscle (IRR = 0.62) and hip/groin injuries (IRR = 0.51) showed lower incidence rates, although the latter was not statistically significant. Reductions were also observed for contact (IRR = 0.71) and non-contact injuries (IRR = 0.73). Subgroup analysis indicated a stronger effect among children (IRR = 0.54) compared with adolescents (IRR = 0.68).

**Conclusions:**

Injury prevention programs are associated with reduced overall and several site-specific injury risks in youth football players. However, evidence for less frequently reported outcomes remains limited and should be interpreted cautiously. Further high-quality, long-term trials are needed to improve the precision of these findings.

## Introduction

Football is one of the most popular sports worldwide, with children and adolescents comprising the largest proportion of participants. Although football offers substantial physical and psychosocial benefits, it is also associated with a relatively high risk of sports-related injuries—particularly among child and adolescent football players who are still undergoing physical development. Due to immature musculoskeletal and neuromotor systems, children and adolescents are more susceptible to injuries during sports participation (Beaudouin et al., 2019; Faude, Rößler & Junge, 2013). The most commonly affected areas include the lower extremities, muscles, ankles, knees, and the hip/groin region, with lower-limb injuries being the most prevalent—occurring at a rate of approximately 6.8 injuries per 1000 h of exposure (López-Valenciano et al., 2020). Previous studies have also shown that injury risk increases with age during adolescence (Obertinca et al., 2023; Rössler et al., 2015), and that the level of biological maturity significantly influences both the type and likelihood of injury.

Biological maturity refers to the stage of physiological and structural development, which may differ considerably from chronological age. During adolescence, rapid increases in stature, body mass, and hormonal activity can transiently impair neuromuscular coordination and balance, thereby increasing susceptibility to injury. Around the period of peak height velocity (PHV)—the phase of maximal growth rate—youth athletes experience pronounced changes in limb length, growth plate vulnerability, and imbalances between muscle and bone strength, all of which elevate the risk of sports injuries (Bult, Barendrecht & Tak, 2018; Le Gall, Carling & Reilly, 2007; Pakarinen, Ponkilainen & Kuitunen, 2025; Parry et al., 2024). These developmental factors suggest that both the risk of injury and the response to prevention programs may differ between children and adolescents, highlighting the importance of age-specific considerations when designing injury prevention interventions.

Several high-quality randomized controlled trials (RCTs) have demonstrated that injury prevention programs can effectively reduce injury incidence among young football players (Al Attar et al., 2023; Beaudouin et al., 2019; Hilska et al., 2021; Owoeye et al., 2014; Waldén et al., 2012; Zarei et al., 2020). However, compared with adult populations, research specifically targeting children and adolescents remains limited. For example, a recent meta-analysis of 44 studies found that injury prevention programs reduced overall injury risk across age and sex groups (RR = 0.71) (Valentin, Linton & Sculthorpe, 2024). Despite its broad scope, the number of studies involving children and adolescent athletes was relatively small, limiting the generalizability of its conclusions for this subgroup. Similarly, Obertinca et al. (2023) evaluated injury prevention programs across all age groups but reported insufficient data focusing specifically on children and adolescents—particularly regarding sex-specific differences and injury-site patterns (*e.g.*, lower-limb and knee injuries). In contrast, the present meta-analysis addresses this gap by including studies that separately report outcomes for children and adolescents across multiple injury sites.

To date, few systematic reviews have comprehensively examined the effectiveness of different injury prevention strategies in child and adolescent populations, particularly concerning program types and site-specific outcomes. Earlier reviews assessing exercise-based injury prevention programs in youth and adult football players have yielded inconsistent results. Roessler et al. (2014) reported that neuromuscular training programs such as FIFA 11+ and FIFA 11+ Kids effectively reduced lower-limb injuries in young players but lacked age-specific subgroup analyses. Similarly, Soomro et al. (2016) found positive effects of prevention programs in adolescent team sports, yet included limited data for younger children. More recently, Obertinca et al. (2023) confirmed the overall benefits of multicomponent programs across footballers of varying ages but did not clarify whether effectiveness differed between children and adolescents.

To address this limitation, the present study systematically evaluates the effectiveness of commonly used injury prevention programs—namely FIFA 11 (Steffen et al., 2008), FIFA 11+ (Owoeye et al., 2014), FIFA 11+ Kids (Al Attar et al., 2023; Rössler et al., 2018; Zarei et al., 2020), structured warm-up routines, and neuromuscular training—on both overall and site-specific injury outcomes among child and adolescent football players using meta-analytic methods. These programs differ in structure and training focus: FIFA 11 emphasizes core stability, balance, plyometric exercises, and strength training; FIFA 11+ expands on this by incorporating dynamic running elements to enhance variability (Thorborg et al., 2017); FIFA 11+ Kids prioritizes single-leg dynamic stability, trunk control, and landing mechanics (Zarei et al., 2020); and neuromuscular training integrates aerobic, strength, balance, and agility components, which can be tailored to the demands of specific sports or populations (Emery et al., 2020).

Although several studies have investigated these programs in child and adolescent populations, their findings remain inconsistent. Therefore, this systematic review and meta-analysis aimed to quantify the effects of injury prevention programs in reducing both overall injury incidence and site-specific injury risks (*e.g.*, lower-limb, knee, and ankle injuries) among child and adolescent football players, by comparing injury rates between intervention and control groups. The findings are expected to provide an evidence-based foundation for optimizing training practices and mitigating injury risks in this vulnerable population.

## Methodology

This systematic review and meta-analysis was conducted in accordance with the Preferred Reporting Items for Systematic Reviews and Meta-Analyses (PRISMA) guidelines (Page et al., 2021). The review protocol was registered in PROSPERO (CRD420251030047; https://www.crd.york.ac.uk/PROSPERO/view/CRD420251030047) on 10 April 2025. The review was initiated on 5 March 2025, indicating that registration occurred after the start of the review and therefore represents retrospective registration. No deviations from the registered protocol were made.

### Search strategy

**Figure 1:**
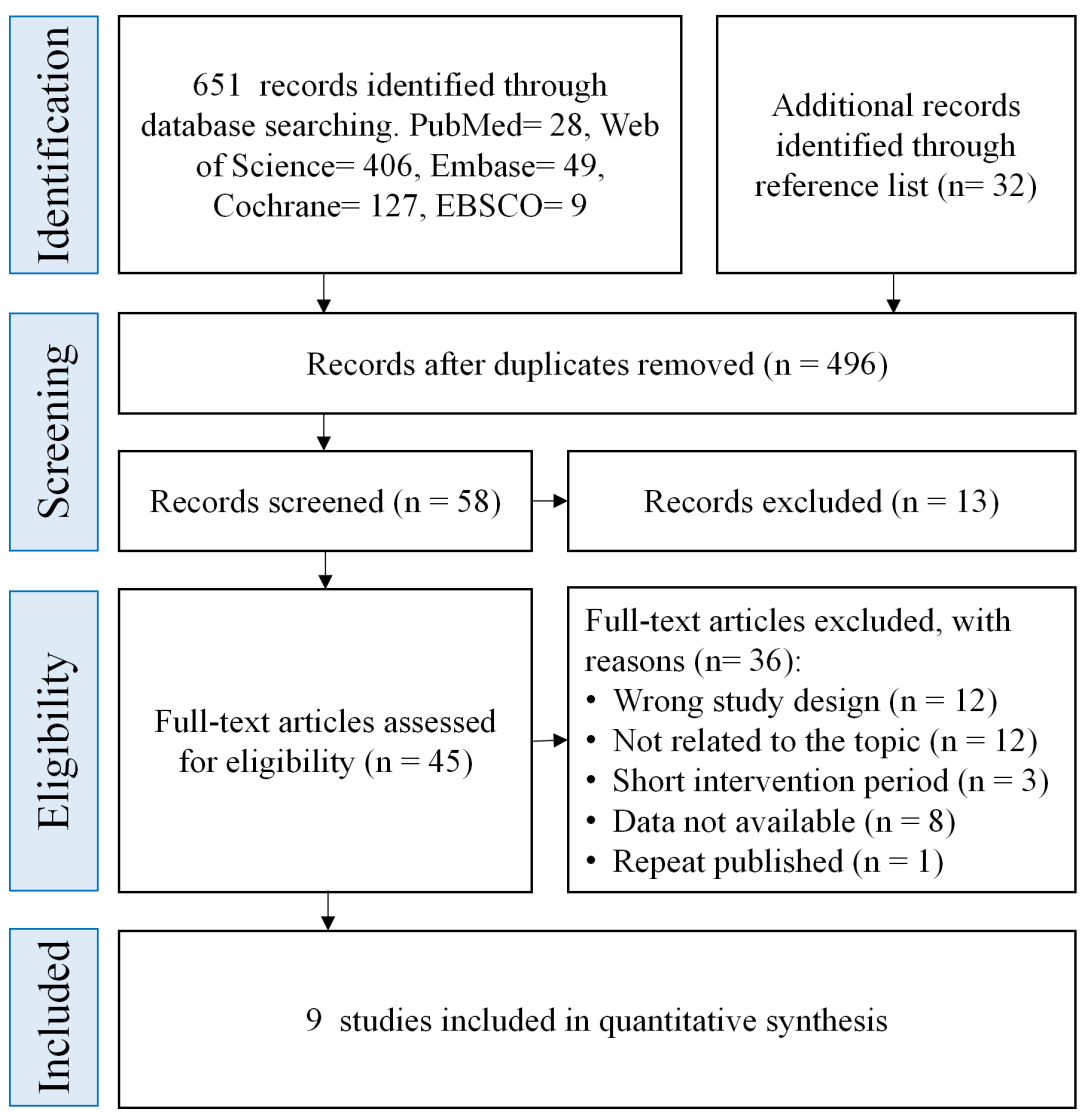
PRISMA 2020 flow diagram of study selection. Each stage in the PRISMA flow diagram illustrates the literature screening process: records identified *via* databases (PubMed, Web of Science, *etc.*) and reference lists are refined by removing duplicates, screening titles/abstracts, and assessing full-texts. Exclusions at each step follow predefined criteria (*e.g.*, wrong study design, irrelevant topics). The final nine studies progress to quantitative synthesis, ensuring transparency in study selection for this meta-analysis.

A systematic search was conducted to identify relevant randomized controlled trials (RCTs) for this meta-analysis. Electronic databases including Cochrane Library, PubMed, EBSCO, Embase, and Web of Science were comprehensively searched without date restrictions, with the final search update completed in March 2026. The following keywords and their combinations were used: “ injury,” “sports injury”,“athletic injury”,“musculoskeletal injury” , “sprain” , “strain” , “ankle injury” , “knee injury” ,“thigh injury” , “hip injury” ,“groin injury”, “pelvic injury”,“adolescents,” “children,” “prevention,” “program,” “football”, “soccer”,“randomized controlled trial”,“cluster randomized controlled trial” ,“FIFA 11,” and “warm-up program.” The study selection process is summarized in the PRISMA flow diagram (Fig. 1). Full search strategies and the data extraction form are provided in the Supplementary Material.

Two reviewers independently screened titles and abstracts, followed by full-text assessment of potentially eligible studies. Disagreements were resolved through discussion, with consultation from a third reviewer when necessary. Inter-rater agreement for study selection was assessed using Cohen’s kappa coefficient, indicating substantial agreement (*k* = 0.68). Data extraction was conducted independently by two reviewers using a standardized data extraction form provided in the Supplementary Material. The form was pilot-tested on a subset of included studies to ensure consistency and clarity prior to full data extraction. Extracted items included author(s), year of publication, country, study design, participant characteristics, intervention details, follow-up duration, exposure time, and outcome measures. Data were extracted from both the main text and Supplementary Material of the original studies, where applicable.

Discrepancies between reviewers were first resolved through discussion to reach consensus; if agreement could not be achieved, a third reviewer acted as an arbitrator. Any disagreements were resolved through discussion with a third reviewer.

### Study selection

Studies were included if they met the following criteria: (1) adopted a randomized controlled trial (RCT) design; (2) implemented an exercise-based injury prevention program (*e.g.*, FIFA 11, FIFA 11+, FIFA 11+ Kids, structured warm-up, neuromuscular, or balance training). Although the initial search strategy included broader health-related terms (*e.g.*, education, sleep, nutrition), only studies incorporating physical training components were retained to ensure comparability and consistency of outcomes; (3) enrolled football players aged 3–18 years who participated in organized football activities at various levels (*e.g.*, school, community, amateur club, or professional academy). When reported age ranges spanned both categories—children (<13 years) and adolescents (13–18 years)—studies were classified according to the predominant age distribution. Specifically, if more than 50.0% of participants fell within one category, the study was assigned to that group. For example, studies with age ranges of 7–14 years (Zarei et al., 2020) and 9–14 years (Hilska et al., 2021) were categorized as children, whereas those with age ranges of 12–17 years (Waldén et al., 2012) and 14–19 years (Owoeye et al., 2014) were considered adolescents when the mean age was below 18. Based on the definitions provided by the World Health Organization (World Health Organization, 2022) and the International Olympic Committee consensus statement on youth athlete development (Bergeron et al., 2015), the term “youth” in this study was replaced with more specific classifications—“children” (<13 years) and “adolescents” (13–18 years). (4) the intervention duration lasted at least eight weeks, as shorter periods were considered insufficient to elicit measurable neuromuscular or biomechanical adaptations; (5) the intervention group received a structured injury prevention program, while the control group performed usual warm-up or standard training routines; (6) the study reported post-intervention injury incidence or sufficient data to calculate it.

The primary outcome was the overall injury incidence, while secondary outcomes included injuries to the lower limb, knee, ankle, muscle, and hip/groin, as well as contact and non-contact injuries.

Studies were excluded if they were non-original publications (reviews, commentaries), non-English articles, had small or incomplete samples, did not focus on child or adolescent populations, lacked relevant outcome measures, involved sports other than football, covered multiple sports, were non-randomized, or constituted grey literature without extractable data.

When essential data were missing or unclear, supplementary information was obtained by cross-referencing citations or conducting targeted database searches to ensure data completeness and accuracy.

### Data extraction

All retrieved references were imported into EndNote X9 (Clarivate Analytics, Philadelphia, PA, USA), and duplicate records were removed. Studies were screened individually based on predefined inclusion and exclusion criteria.

Two independent reviewers conducted the risk-of-bias assessment, and disagreements were resolved through discussion or consultation with a third reviewer. Inter-rater agreement for risk-of-bias assessment was evaluated using Cohen’s kappa coefficient, indicating substantial agreement (*k* = 0.74).

In addition to the standard extraction fields, this review incorporated subgroup data related to contact *versus* non-contact injuries and age-based stratification (children <13 years and adolescents 13–18 years). Data extraction was performed independently by two reviewers and cross-checked for accuracy, with discrepancies resolved by a third reviewer (Yang), who made the final decision.

### Quality assessment

The methodological quality of the included studies was systematically evaluated using the Cochrane Risk of Bias tool as outlined in the Cochrane Handbook for Systematic Reviews of Interventions (Higgins et al., 2011). The assessment criteria included: random sequence generation, allocation concealment, blinding of outcome assessment, completeness of outcome data, selective reporting, and other sources of potential bias.

Given the practical challenges of blinding participants and personnel in sports intervention studies, the domain of “blinding of participants and personnel” was not assessed in this review. Blinding of participants and personnel was considered infeasible due to the nature of exercise-based interventions. However, blinding of outcome assessment was evaluated as a separate domain when reported (*e.g.*, blinded injury classification or independent outcome adjudication). When insufficient information was provided, the domain was rated as unclear risk of bias. Each domain was rated as having either a “low risk,” “high risk,” or “unclear risk” of bias.

**Figure 2:**
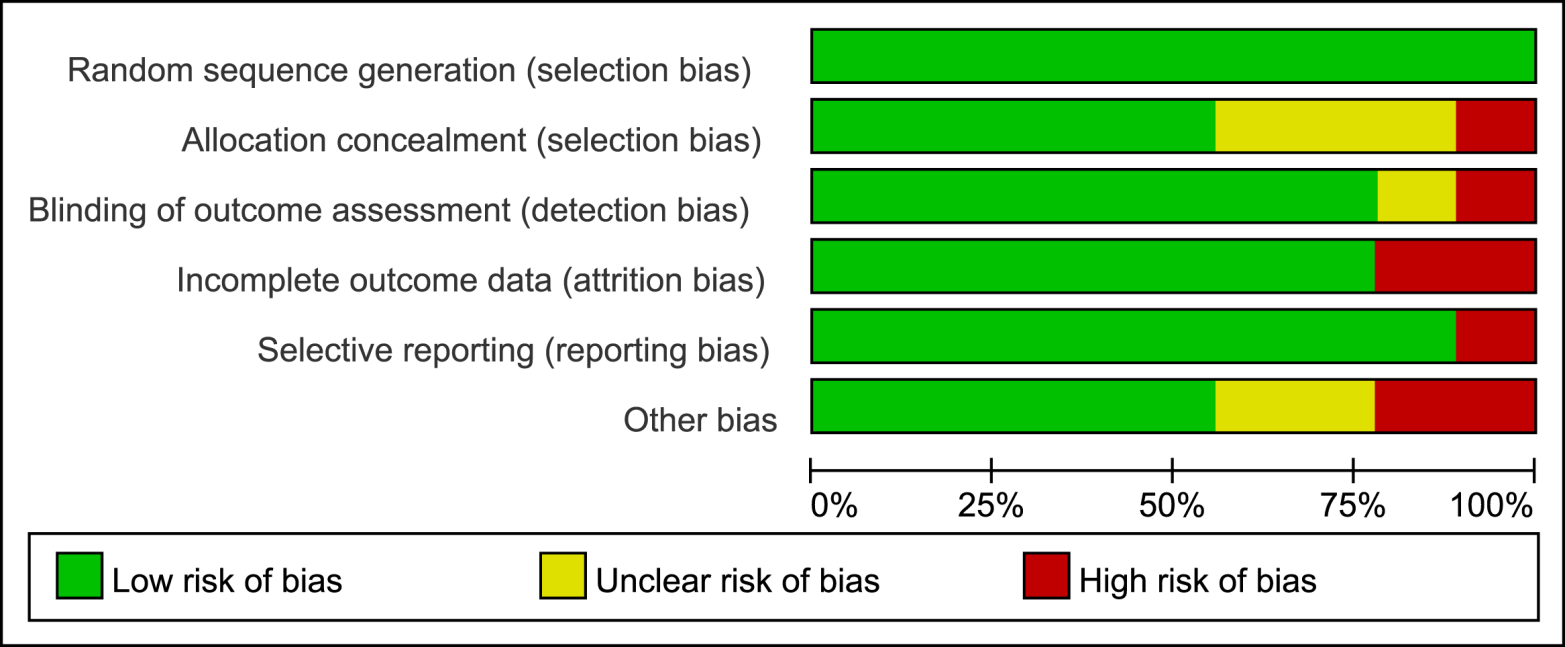
Risk of bias distribution across different assessment domains in the study. Bar chart summarizing risk of bias across six domains: randomization, allocation concealment, blinding, incomplete data, selective reporting, and other bias. Green, low risk; yellow, unclear; red, high risk.

**Figure 3:**
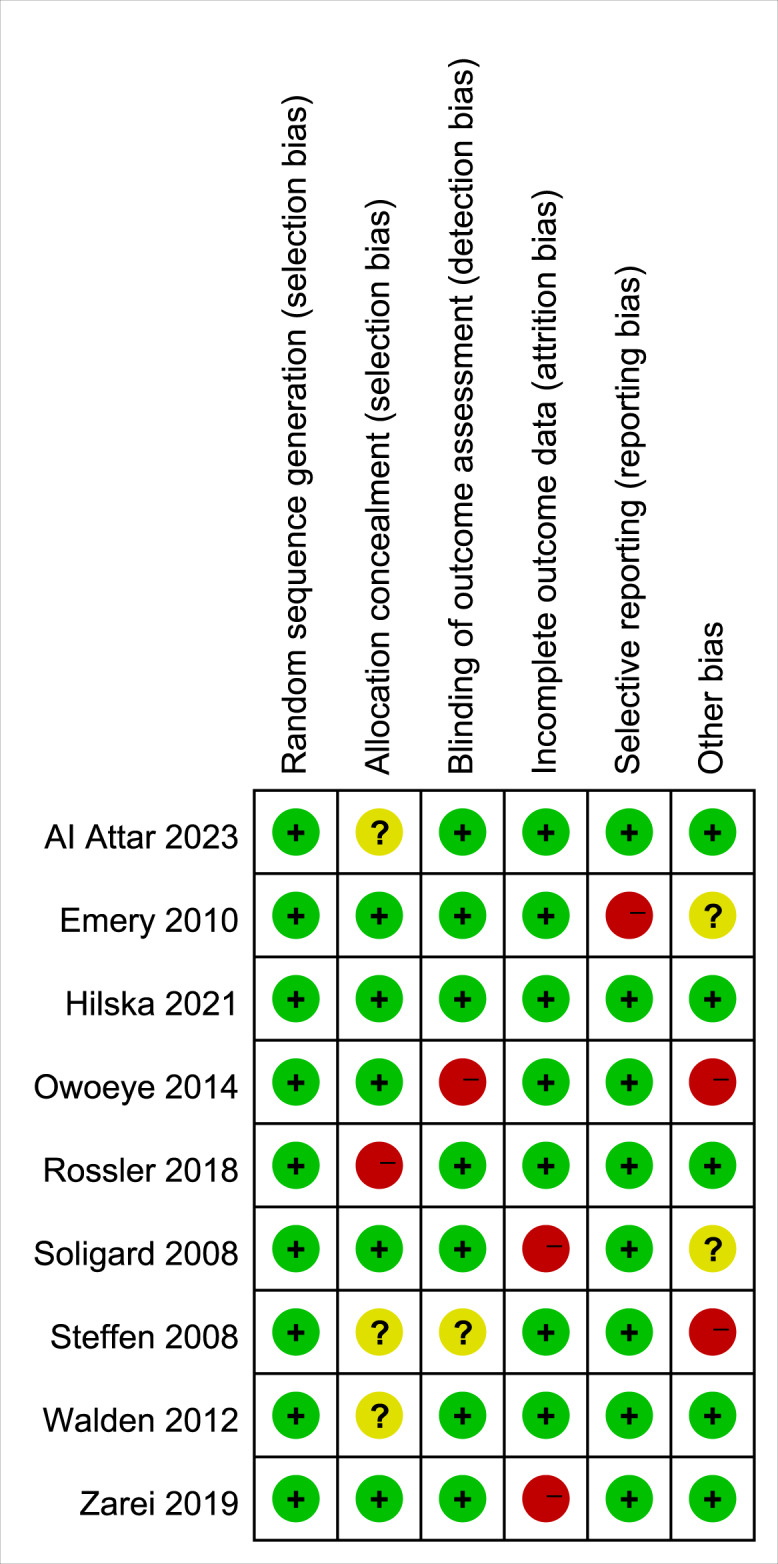
Risk of bias assessment across studies for lower limb injury research. The matrix displays bias risk evaluations for nine studies (Al Attar et al., 2023; Emery & Meeuwisse, 2010, *etc.*) across six domains: Random sequence generation, Allocation concealment, *etc*. Green “+” = Low risk, Yellow “?” = Unclear risk, Red “-” = High risk, showing domain-specific bias risks per study.

Two independent reviewers (Ding and Fan) conducted the assessment, and any disagreements were resolved through discussion or consultation with a third reviewer. A visual summary of the risk of bias is presented in Figs. 2 and 3. Additionally, sensitivity analyses and publication bias assessments were performed using Stata 17.0 (StataCorp LLC, College Station, TX, USA).

### Data synthesis

The incidence rate ratio (IRR) was determined by dividing the injury incidence rate in the intervention group by that in the control group. Specifically, the injury incidence rate for each group was calculated as the number of recorded injury events divided by the total exposure time. Therefore, the IRR represents the ratio of the injury rate in the intervention group to the injury rate in the control group.

In this study, all included studies reported injury incidence based on exposure time (hours or player-hours). Therefore, no conversion from athlete-exposures to exposure hours was required, and all incidence rate ratios (IRRs) were calculated using the original hours-based denominators as reported in the primary studies (Crossley et al., 2020; Obertinca et al., 2023; Su et al., 2025).

In addition to the overall injury risk, secondary outcomes included injuries to the lower limb, knee, ankle, muscle, and hip/groin, as well as contact and non-contact injuries. Given that multiple secondary outcomes were examined, the potential for inflated type I error due to multiple comparisons was acknowledged. The overall injury incidence was prespecified as the primary outcome, while analyses of site-specific and injury-type outcomes were considered secondary and exploratory in nature. Therefore, *p* values for secondary outcomes were interpreted cautiously, with greater emphasis placed on effect sizes and confidence intervals rather than strict statistical significance.

All statistical analyses were performed using Stata 17.0 (StataCorp LLC, College Station, TX, USA). Given the expected clinical and methodological heterogeneity across studies (*e.g.*, intervention content, participant characteristics, and follow-up duration), random-effects models were applied for all meta-analyses regardless of the magnitude of I^2^. The DerSimonian–Laird method was used as the primary estimator of between-study variance (*τ*^2^). Pooled IRRs with corresponding 95.0% confidence intervals (CIs) were estimated using the metan command with the DerSimonian–Laird method as the primary estimator of between-study variance (*τ*^2^). Between-study heterogeneity was assessed using the I^2^ statistic, *τ*^2^, and Cochran’s *Q* test. RevMan 5.3 (The Cochrane Collaboration, Copenhagen, Denmark) was used solely for risk of bias assessment.

Random-effects meta-analyses were conducted in Stata 17.0 (StataCorp LLC, College Station, TX, USA) using the metan command, with the DerSimonian–Laird method as the primary estimator of between-study variance. The potential impact of alternative estimators, such as the Hartung–Knapp–Sidik–Jonkman method, was considered when interpreting results, particularly for outcomes with a small number of studies or substantial heterogeneity.

Publication bias and sensitivity analyses were also conducted in Stata 17.0 (StataCorp LLC, College Station, TX, USA) to evaluate the robustness and reliability of pooled estimates. Egger’s regression test and Begg’s rank correlation test were used to assess potential publication bias. *Post-hoc* power and minimal detectable effect size (MDES) analyses were also performed.

For each pooled outcome, we derived the standard error (SE) of the log incidence rate ratio (lnIRR) from the 95.0% confidence interval (CI) as SE = [ln(upper CI) − ln(lower CI)]/(2 × 1.96). *Post-hoc* power (two-sided *α* = 0.05) was estimated assuming the true effect equals the observed pooled lnIRR using a normal approximation. The minimal detectable effect size at 80.0% power was calculated as —lnIRR—min = (z0.975 + z0.80)×SE and transformed back to the IRR scale. Outcomes with power <0.80 were considered potentially underpowered. Results are provided in Table S3.

### Code availability

The Stata do-file(s) used to reproduce the meta-analyses and figures are provided in File S1. The scripts include commands to (i) generate forest plots (metan), (ii) conduct leave-one-out sensitivity analyses (metaninf) and export high-resolution plots, (iii) produce funnel plots (metafunnel), (iv) assess small-study effects/publication bias (metabias for Egger’s test; meta bias, begg after meta set), and (v) prepare subgroup indicators (children *vs.* adolescents) based on study labels. RevMan 5.3 (The Cochrane Collaboration, Copenhagen, Denmark) was used only for risk of bias assessment and figure generation; domain-level judgments (low risk/high risk/unclear risk) and rationales were entered in the “Support for judgement” field, and the risk-of-bias summary and graph figures were exported directly from RevMan 5.3 (The Cochrane Collaboration, Copenhagen, Denmark).

## Results

### Descriptive analysis of the identified studies

A total of 651 potentially relevant articles were initially retrieved from the electronic databases. An additional 32 articles were identified through reference list screening. After removing duplicates, 496 unique records remained. Based on titles and abstracts, studies that clearly failed to meet the inclusion criteria were excluded, leaving 45 articles for full-text review. After further screening, 9 randomized controlled trials (RCTs) met the eligibility criteria and were ultimately included in the meta-analysis. Reasons for exclusion included: irrelevant outcomes, lack of injury-related data, non-randomized designs, interventions not specific to football, or study populations not limited to children and adolescents. The detailed study selection process is illustrated in Fig. 1.

### Sample characteristics

Based on the data extracted from the included studies, a total of 16,636 participants were enrolled, all of whom were male or female youth football players aged between 3 and 18 years. Across all studies, a total of 1,711 overall injury cases were reported, with 701 injuries occurring in the intervention groups and 1,010 in the control groups. In addition, a total of 4,624 injuries were documented for specific body regions (including lower limbs, ankles, knees, hips/groin, and muscles) and injury types (contact and non-contact).

Regarding the intervention protocols, one study implemented FIFA 11+ (Owoeye et al., 2014). One study evaluated “The 11” (Soligard et al., 2008), three studies employed the FIFA 11+ Kids protocol (Al Attar et al., 2023; Rössler et al., 2018; Zarei et al., 2020), and another three studies utilized neuromuscular training (NMT) programs (Emery & Meeuwisse, 2010; Matias et al., 2021; Waldén et al., 2012). One study adopted a structured FIFA 11 warm-up protocol (Steffen et al., 2008). Detailed study characteristics are presented in Tables 1 and 2. Full search strategies are provided in Table S1, and the data extraction form is provided in Table S2.

**Table 1:** Characteristics of soccer injury prevention studies: country, population, intervention, and sample details.

**Study**	**Country**	**Population** **(all soccer)**	**Age classification**	**Study design**	**Follow-up**	**Intervention program**	**Intervention components**	**Sample size analysed**	**Outcome measure**	**Main outcomes after intervention**	**Compliance/ adherence**
Al Attar et al. (2023)	Saudi Arabia	Male children (7–13 years)	Children	Cluster RCT	6 months	FIFA 11+ Kids	Dynamic warm-up, balance, plyometric, coordination	IG: 377 people CG: 363 people	Overall injury rate	↓Injury rate by 57.0% IRR = 0.43	NR
Zarei et al. (2020)	Iran	Male children (7–14 years)	Children (majority <13)	RCT	9 months	FIFA11+ Kids	Strength, balance, agility	IG: 443 people CG: 519 people	Overall injury rate	↓Total injuries by 50.0% IRR = 0.50	NR
Hilska et al. (2021)	Finland	Male and female children (9–14 years)	Children (majority <13)	RCT	20 weeks	Neuromuscular training (NMT) warm - up	Balance, core, coordination	IG: 673 people CG: 730 people	Injury incidence per 1000 h	↓Knee injury rate by 25.0%; IRR = 0.75	NR
Owoeye et al. (2014)	Nigeria	Male youth (14–19 years)	Adolescent (mean = 15.8)	Cluster RCT	6 months	FIFA 11 +	Warm-up, strength, agility	IG: 212 people CG: 204 people	Overall injury rate	↓Overall injury rate by 54.0% (IRR = 0.46; *p* < 0.05)	Training attendance reported
Waldén et al. (2012)	Sweden	Female youth (12–17 years)	Adolescent	Cluster RCT	7 months	Neuromuscular warm - up program	Core stability, jump/landing, balance	IG: 2,479 people CG: 2,085 people	Knee injury rate	↓ACL injuries by 64.0% IRR = 0.36	NR
Emery & Meeuwisse (2010)	Canada	Male and female youth (13–18 years)	Adolescent	Cluster RCT	20 weeks	Neuromuscular training	Core, balance, plyometric	IG: 380 people CG: 364 people	Injury risk ratio	↓Injury risk (IRR = 0.62; 95.0% CI [0.39–0.99])	NR
Steffen et al. (2008)	Norway	Female youth (13–17 years)	Adolescent	Cluster RCT	8 months	FIFA 11	Warm-up, balance, running drills	IG: 1,091 people CG: 1,001 people	Overall injury rate	no obvious decline IRR = 0.99	Reported (sessions per week); not quantified
Rössler et al. (2018)	Switzerland, Germany, Czech Republic, The Netherlands	Male and female children (7–13 years)	Children	Cluster RCT	9 months	FIFA 11+ Kids	Warm-up, strength, coordination	IG: 2,066 people CG: 1,829 people	Overall injury rate	↓Overall injury rate by 50.0% Injury risk IRR = 0.50	Compliance monitored; quantitative rate not reported
Soligard et al. (2008)	Norway	Female youth (13–17 years)	Adolescent	Cluster RCT	8 months	The 11	Strength, balance, running technique	IG: 1,055 people CG: 837 people	Injury risk ratio	↓Overall injury rate by 32.0% IRR = 0.68	Reported; not quantified

**Notes.**

Table lists 9 soccer - related studies (*e.g.*, Al Attar et al., 2023; Zarei et al., 2020). Columns show study name, country, population (age/range, gender), follow - up duration, intervention program (*e.g.*, FIFA 11 +, Neuromuscular training), and sample sizes (IG, Intervention Group; CG, Control Group).

NR not reported IG intervention group CG control group H hours IRR incidence rate ratio

Injury incidence rates were calculated per 1000 exposure hours.

**Table 2:** Injury incidence across soccer intervention studies: body region and group comparisons.

**Study**	**Intervention**	**Participants** **analysed**	**Exposure hours**	**Overall** **injuries**	**Lower limb injuries**	**Knee injuries**	**Ankle** **injuries**	**Hip/Groin** **injuries**	**Muscle injuries**	**Contact** **injury**	**Nno-contact injury**
Zarei et al. (2020)	FIFA11+ Kids	IG:443people CG:519 people	IG:31,934.00H CG:32,113.00H	IG:30.00 CG:60.00	IG:24.00 CG:54.00	IG:6.00 CG:18.00	IG:9.00 CG:16.00	NR	NR	NR	NR
Hilska et al. (2021)	Neuromuscular training (NMT) warm - up	IG:673 people CG:730 people	NR	NR	IG:310.00 CG: 340.00	IG:21.00 CG:25.00	IG:40.00 CG:60.00	NR	IG: 57.00 CG:77.00	IG: 181.00 CG:173.00	IG: 129.00 CG:173.00
Owoeye et al. (2014)	FIFA 11 +	IG:212 people CG:204 people	IG: 51,017 .00H CG:61,045.00H	IG:36.00 CG:94.00	IG:26.00 CG:76.00	NR	IG:12.00 CG:21.00	NR	NR	IG: 27 CG: 66	IG: 7.00 CG: 16.00
Waldén et al. (2012)	Neuromuscular warm-up program	IG:2,479 people CG:2,085 people	IG:14,921.40H CG:12,908.40H	NR	NR	IG:7.00 CG:14.00	NR	NR	NR	NR	NR
Emery & Meeuwisse (2010)	Neuromuscular training	IG:380 people CG:364 people	IG:24,051.00 H CG:23,597.00H	IG:50.00 CG:79.00	IG:42.00 CG:60.00	IG:3.00 CG:8.00	IG:14.00 CG:27.00	NR	NR	NR	NR
Steffen et al. (2008)	FIFA 11	IG:1,073 people CG:947 people	IG:66,423.00 H CG:65,725.00H	IG:242.00 CG:241.00	IG:181.00 CG:173.00	IG:37.00 CG:30.00	IG:79.00 CG:74.00	IG:6.00 CG:14.00	IG:45.00 CG:42.0 0	IG:118.00 CG:124.00	IG:93.00 CG:86.00
Soligard et al. (2008)	The 11	IG:1,055 people CG:837 people	IG:49,899.00 H CG:45,428.00H	IG:161.00 CG: 215.00	IG:121.00 CG:143.00	IG: 35.00 CG: 58.00	IG: 51.00 CG: 52.00	IG:10.00 CG:9.00	IG:5.00 CG:8.00	IG:53.00 CG:76.00	IG:55.00 CG:58.00
Al Attar et al. (2023)	FIFA 11+ Kids	IG:377 people CG: 363 people	IG: 50,120.00H, CG: 42,616.00H	IG: 43.00 CG: 86.00	IG:26.00 CG:42.00	IG: 9.00, CG: 18.00	IG: 6.00, CG: 14.00	NR	IG: 8.00 CG: 16.00	IG:26.00 CG:44.00	IG: 12.00 CG: 26.00
Rössler et al. (2018)	FIFA 11+ Kids	IG:2,066 people CG:1,829 people	IG: 14,071.60 H CG: 15,203.30 H	IG: 139.00 CG: 235.00	IG: 100.00 CG: 170.00	IG: 29.00 CG: 54.00	IG: 26.00 CG: 44.00	IG: 4.00 CG: 16.00	NR	NR	NR

**Notes.**

Continued table of characteristics of included studies (including comparison of injury numbers in general, lower limb, knee joint, ankle joint, muscle, hip joint and groin).

Details injury data for soccer studies (*e.g.*, Zarei et al., 2020; Al Attar et al., 2023). Columns: Study name, intervention, participant groups (IG, Intervention Group, CG, Control Group), exposure hours, and injury counts by region (Overall, Lower limb, Knee, Ankle, Hip/Groin, Muscle).

NR not reported NG No Group - specific Data IG intervention group CG control group H hours

Injury data from the included studies were collected using standardized tools, as described in Table S4. Detailed inter-rater agreement data for study selection and risk-of-bias assessment are provided in Table S5. Injury definitions followed the time-loss criterion by Fuller et al. (2006), classifying injuries by severity and body region (*e.g.*, lower extremities, knee, ankle). Data collection was performed weekly *via* injury report forms and structured interviews, ensuring consistent injury classification across studies, the injury surveillance tools employed (*e.g.*, IRFs, web-based platforms) (Emery, Meeuwisse & Hartmann, 2005) ensured consistent and reliable injury data collection across studies. These tools allowed for standardized injury definitions and classification, ensuring the comparability of injury outcomes across studies and intervention types.

### Meta-analysis results

A total of seven randomized controlled trials were included in the meta-analysis of overall injury incidence, while two studies were excluded because they did not report relevant overall injury outcomes. For the primary outcome, sensitivity analysis using the Hartung–Knapp–Sidik–Jonkman (HKSJ) method yielded an identical pooled estimate (IRR = 0.62, 95% CI [0.47–0.81]) compared with the DerSimonian–Laird model (IRR = 0.62, 95% CI [0.49–0.78]).The corresponding forest plot is shown in Fig. S1. Although the confidence interval was slightly wider, the effect remained statistically significant, confirming the robustness of the findings.

*Post-hoc* power analyses suggested that several outcomes (*e.g.*, hip/groin, contact, non-contact, and the adolescents subgroup) were underpowered (power <0.80; Table S3), whereas the primary outcome and some major site-specific outcomes were adequately powered.

Among seven RCTs analyzing overall injuries, intervention groups (*n* = 5,606) experienced 675 injuries, whereas the control groups included 5,603 participants with 896 injury events.

Using the random-effects model, the pooled analysis demonstrated that the intervention groups had a significantly lower risk of overall injury compared with the controls, with an incidence rate ratio (IRR) of 0.62 (95.0% CI [0.49–0.78], *p* < 0.001).

**Figure 4:**
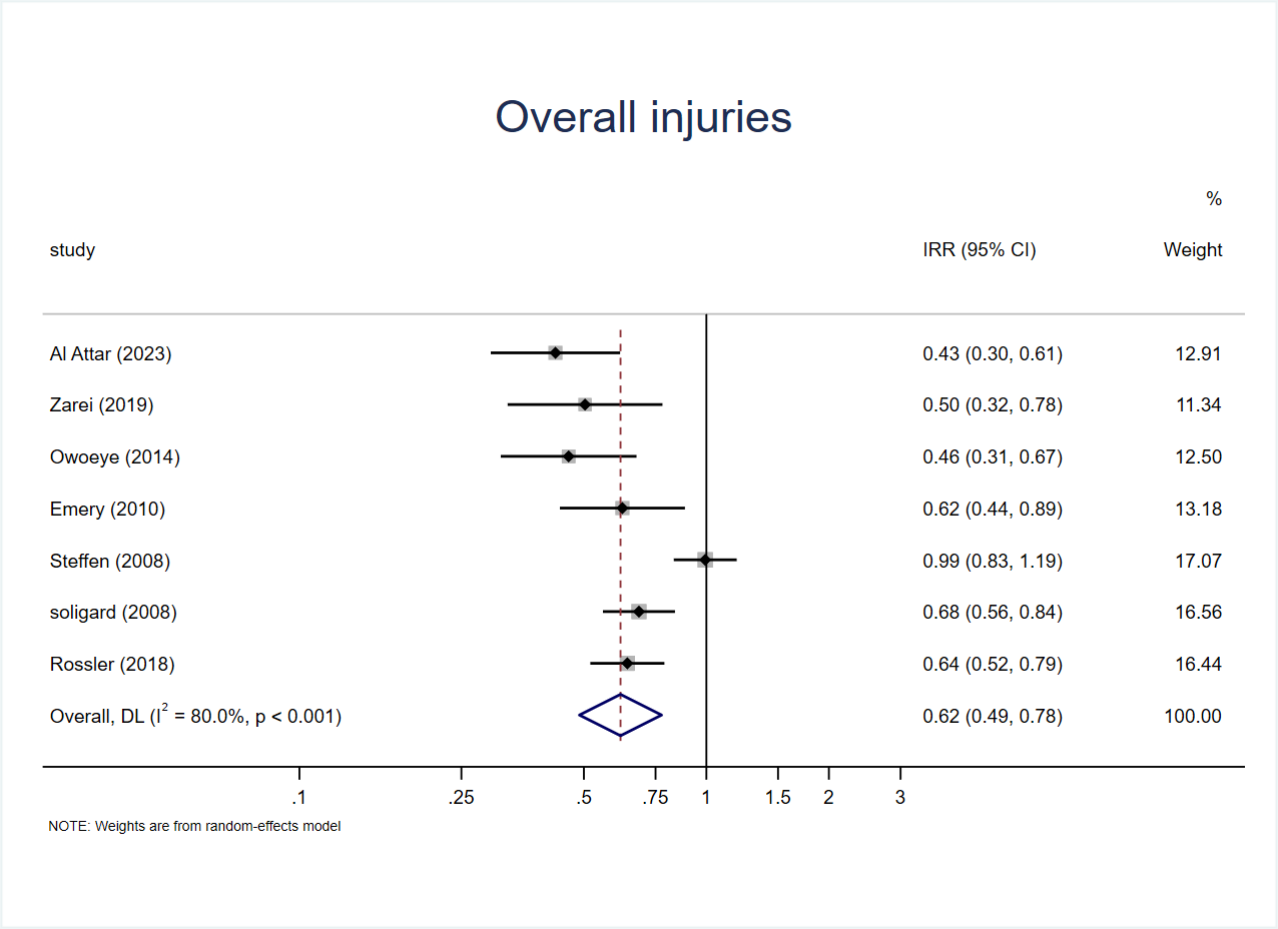
Forest plot of overall injury incidence rate ratios (IRR) among youth football players. Squares represent study-specific IRRs (size proportional to study weight) and horizontal lines indicate 95% CIs. The diamond denotes the pooled IRR, with its width representing the 95% CI. The vertical dashed line at IRR = 1.0 indicates no effect; values to the left (IRR < 1.0) favor the intervention. I^2^ and the heterogeneity-test *p* value are reported in the plot.

This finding corresponds to an approximate 38.0% reduction in overall injury risk per 1000 exposure hours (in Fig. 4).

However, a high degree of heterogeneity was observed among the included studies (*I*^2^ = 80.0%, *p* < 0.001), indicating considerable variation across studies, possibly related to differences in intervention content, training intensity, or adherence levels.

Regarding secondary outcomes, the meta-analysis of lower limb injuries revealed a pooled IRR of 0.68 (95.0% CI [0.56–0.82]), suggesting that the injury prevention programs also conferred a significant protective effect on lower limb injuries in Fig. S2.

The heterogeneity analysis showed *I*^2^ = 72.4%, indicating substantial variability among studies. This heterogeneity may stem from differences in the type and duration of interventions, participant compliance, and variations in age distribution among the study populations.

### Knee injuries

All included studies reported knee injury outcomes, encompassing a total of 16,636 players. As shown in Fig. S3, the pooled analysis yielded an incidence rate ratio (IRR) of 0.67 (95.0% CI [0.52–0.87]; *p* = 0.08). The heterogeneity was relatively low (*I*^2^ = 42.9%), indicating good consistency among studies. These findings suggest that structured injury prevention programs provide a moderate but meaningful protective effect against knee injuries in child and adolescent football players.

### Ankle injuries

Among the nine included studies, eight reported data related to ankle injuries, involving a total of 12,072 players. As shown in Fig. S4, the pooled analysis yielded an incidence rate ratio (IRR) of 0.72 (95.0% CI [0.61–0.86]; *p* = 0.05). The heterogeneity test indicated a moderate level of heterogeneity (*I*^2^ = 49.7%), suggesting partial variability across studies in terms of intervention design and implementation. Overall, the results indicate that injury prevention programs exert a preventive effect on ankle injuries among child and adolescent football players, although the strength of this effect appears to be moderate.

### Muscle injuries

Three studies reported data on muscle injuries, involving a total of 4,035 child and adolescent football players. As shown in Fig. S5, the pooled analysis yielded an incidence rate ratio (IRR) of 0.62 (95.0% CI [0.46–0.84]; *p* = 0.63; *I*^2^ = 0.00%), indicating excellent consistency among the included studies. These results suggest that injury prevention programs confer a modest yet consistent protective effect against muscle injuries in young football players.

### Hip and groin injuries

Three of the nine included studies reported data on hip and groin injuries, As shown in Fig. S6 the pooled estimate suggested a potential reduction in the intervention group; however, this effect was not statistically significant (IRR = 0.51, 95.0% CI [0.24–1.09]). The wide confidence interval indicates substantial imprecision, and the finding should be interpreted as a trend rather than definitive evidence of an effect.

### Contact injuries

Out of the nine included studies, five reported outcomes related to contact injuries, involving a total of 888 players. As shown in Fig. S7, the pooled analysis yielded an incidence rate ratio (IRR) of 0.71 (95.0% CI [0.55–0.93]; *p* < 0.001; *I*^2^ = 70.5%), indicating a relatively high level of heterogeneity. These findings suggest that injury prevention programs exert a moderate protective effect in reducing contact injuries among child and adolescent football players, supporting their practical applicability in real-world training and competition settings.Given the limited sample size/number of trials, substantial heterogeneity, and underpowered analysis (power <0.80; Table S3), this finding should be considered exploratory rather than a definitive estimate.

### Non-contact injuries

Five studies reported data on non-contact injuries, including a total of 655 players. As shown in Fig. S8, the pooled analysis yielded an incidence rate ratio (IRR) of 0.73 (95.0% CI [0.54–0.99]; *p* = 0.02; *I*^2^ = 64.3%), indicating a relatively high level of heterogeneity. These results suggest that injury prevention programs exert a weaker but still notable preventive effect on non-contact injuries among child and adolescent football players.Given the limited sample size/number of trials, substantial heterogeneity, and underpowered analysis (power <0.80; Table S3), this analysis should be considered exploratory rather than a definitive estimate.

### Heterogeneity and sensitivity analyses

Sensitivity analyses were performed for the four outcomes with the largest number of included studies—overall injuries, lower limb injuries, ankle injuries, and knee injuries—to evaluate the influence of individual studies on the pooled results and heterogeneity (see File S1).

For overall injuries, the study by Steffen et al. (2008) showed a marked deviation from the other included trials. After excluding this study, the pooled incidence rate ratio (IRR) decreased from 0.62 to 0.58, and the heterogeneity (I^2^) was substantially reduced from 80.0% to 36.0%, indicating that this study might have been a major contributor to the high heterogeneity observed in the overall analysis.

Similarly, for lower limb injuries, the study by Steffen et al. (2008) exhibited considerable deviation. Excluding this study reduced the pooled IRR from 0.68 to 0.59, suggesting that it may have been another potential source of heterogeneity.

In contrast, the sensitivity analyses for ankle and knee injuries demonstrated high robustness. Sequential exclusion of individual studies produced only minimal changes in the pooled effect sizes, indicating that these outcomes were less influenced by any single study and that the meta-analytic results were stable and reliable.

Overall, variations in sample characteristics, intervention protocols, or compliance levels across studies are likely key contributors to the observed heterogeneity and should be carefully considered in the interpretation of results.

### Publication bias assessment

Because some outcomes included a limited number of studies, publication bias was assessed only for those with at least seven RCTs, namely overall injuries, lower limb injuries, ankle injuries, and knee injuries. Publication bias was visually examined using funnel plots and statistically tested using Egger’s regression and Begg’s rank correlation tests in Figs. S9–S12.

For overall injuries, the funnel plot showed slight asymmetry. Egger’s test yielded *p* = 0.03 (<0.05), whereas Begg’s test yielded *p* = 0.07 (>0.05), suggesting a possible small degree of publication bias. The trim-and-fill analysis identified no missing studies (k_0_ = 0), and the pooled incidence rate ratio (IRR) remained unchanged from the main analysis, indicating that the overall results were robust. Nevertheless, these findings should be interpreted cautiously due to the limited number of studies available.

For ankle injuries, the funnel plot appeared moderately asymmetric. Egger’s test indicated potential publication bias (*p* = 0.01), and Begg’s test also suggested possible small-study effects (*p* = 0.04). After applying the trim-and-fill method, four hypothetical studies were imputed, increasing the pooled IRR from 0.72 to 0.82 (95.0% CI [0.70–0.96]). This suggests that the original effect size may have been slightly overestimated. Given the limited number of included trials, these results should be interpreted cautiously.

In contrast, the funnel plot for knee injuries was nearly symmetrical. Egger’s (*p* = 0.15) and Begg’s (*p* = 0.25) tests both indicated the absence of significant publication bias, supporting the reliability of the pooled estimate.

For lower limb injuries, the funnel plot showed mild asymmetry. Egger’s test yielded *p* = 0.03, suggesting possible publication bias, and Begg’s test also suggested possible small-study effects (*p* = 0.04).The trim-and-fill procedure added three hypothetical studies, increasing the pooled IRR from 0.68 to 0.78, implying that the original pooled estimate might have been slightly inflated.

Overall, except for ankle and lower limb injury outcomes where potential bias was detected, the remaining analyses demonstrated good stability. Even after trim-and-fill adjustment, the pooled IRRs retained statistical significance, confirming the robustness and validity of the main findings.

### Subgroup analysis: children *vs.* adolescents

For the overall injury outcome, a subgroup analysis was conducted to compare the effectiveness of intervention programs between different age groups—children (<13 years) and adolescents (13–18 years).

In the children’s subgroup, the pooled incidence rate ratio (IRR) was 0.54 (95.0% CI [0.41–0.70]; *I*^2^ = 48.8%, *p* = 0.14), indicating relatively low heterogeneity and a stable, reliable preventive effect.

**Figure 5:**
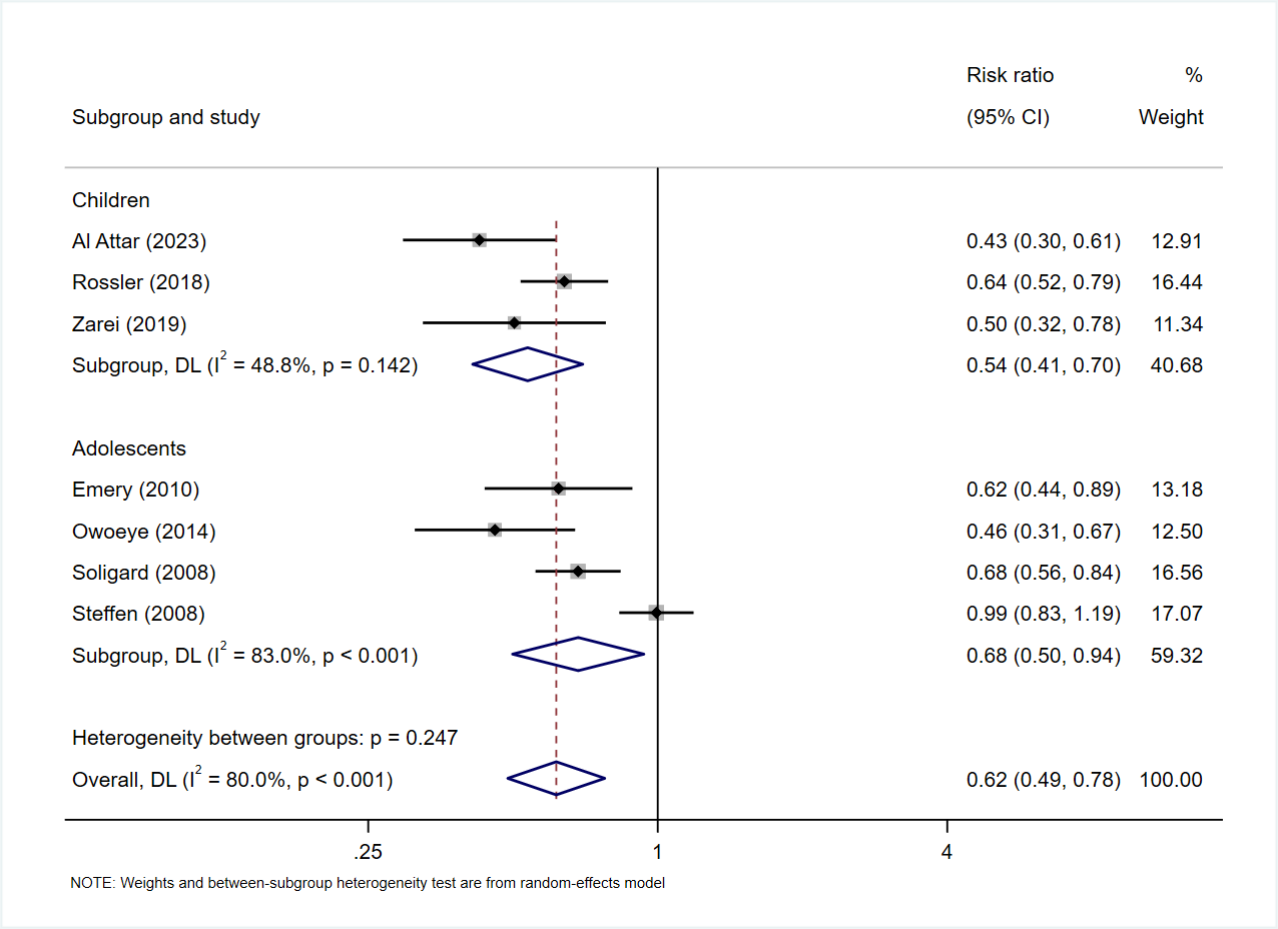
Subgroup meta-analysis of injury incidence rate ratios (IRR) by age group (children *vs.* adolescents). Squares represent study-specific IRRs (size proportional to study weight) and horizontal lines indicate 95% CIs. The diamond denotes the pooled IRR, with its width representing the 95% CI. The vertical dashed line at IRR = 1.0 indicates no effect; values to the left (IRR < 1.0) favor the intervention. I^2^ and the heterogeneity-test *p* value are reported in the plot.

In the adolescent subgroup, the pooled IRR was 0.68 (95.0% CI [0.50–0.94]; *I*^2^ = 83.0%, *p* < 0.001), suggesting a higher degree of heterogeneity, possibly attributable to differences in training background, intervention content, compliance levels, or stages of physiological development (see Fig. 5).

Overall, both subgroups demonstrated significant injury-prevention benefits, with the intervention effect being slightly stronger and more consistent among children than adolescents, indicating that younger players may respond more uniformly to structured injury prevention programs.

## Discussion

### Summary of main findings

This meta-analysis included nine randomized controlled trials that evaluated the effectiveness of injury prevention programs in reducing overall and site-specific injury incidence among child and adolescent football players. It is the first to perform an age-stratified meta-analysis. The subgroup analysis showed a stronger preventive effect in children (IRR = 0.54, 95.0% CI [0.41–0.70]) than in adolescents (IRR = 0.68, 95.0% CI [0.50–0.94]), with lower heterogeneity among children (*I*^2^ = 48.8%) than adolescents (*I*^2^ = 83.0%), suggesting more consistent responses among younger players.

The greater benefits observed in children may relate to biological maturity, exposure, and neuromuscular adaptability. Prepubertal players (<13 years) experience more stable growth and lower training loads, enhancing their response to neuromuscular and balance-based interventions. In contrast, adolescents (13–18 years) undergo rapid changes during the peak height velocity (PHV) phase, which may transiently impair coordination and increase injury susceptibility (Le Gall, Carling & Reilly, 2007; Pakarinen, Ponkilainen & Kuitunen, 2025). These findings highlight developmental differences and the importance of age-tailored prevention strategies.

Overall, programs such as FIFA 11+, structured warm-ups, and neuromuscular training significantly reduced injury risk, with a pooled IRR of 0.62 (95.0% CI [0.49–0.78]), corresponding to a 38.0% decrease in injury incidence per 1000 exposure hours. For site-specific outcomes, children showed stronger preventive effects for knee (IRR = 0.53) and ankle (IRR = 0.54) injuries than adolescents (IRR = 0.75 and 0.71), possibly due to higher motor plasticity and lower cumulative load. Both age groups benefited from reduced contact (IRR = 0.71) and non-contact (IRR = 0.73) injuries, indicating broad preventive efficacy across mechanisms.

Although heterogeneity was higher among adolescents, likely reflecting differences in maturity, competition exposure, and compliance, the general trend supports the robust effectiveness of structured prevention programs. After trim-and-fill adjustment, effect sizes for lower-limb and ankle injuries remained significant, reinforcing the stability of the findings.

In summary, injury prevention programs confer strong protective benefits in youth football, with children exhibiting greater and more consistent effects—particularly at the knee and ankle. Evidence for hip/groin injuries remains uncertain due to the limited number of trials and wide confidence intervals; therefore, any age-related differences for this outcome should be considered hypothesis-generating rather than conclusive. These results emphasize tailoring interventions to developmental stage: focusing on coordination and balance in younger players, and progressive strength and load management in older adolescents to optimize long-term safety and performance.

### Interpretation of results and mechanistic insights

The findings of this meta-analysis suggest that the observed injury prevention effects of the intervention programs may arise from multiple underlying mechanisms. Neuromuscular training (NMT) has been widely shown to enhance balance, postural control, and core stability—key factors in reducing sports-related injuries (Su et al., 2025).

Similarly, FIFA-based injury prevention programs—including FIFA 11, FIFA 11+, and FIFA 11+ Kids—employ a multidimensional warm-up framework integrating dynamic stretching, agility and balance drills, plyometric exercises, and progressive core-strengthening routines.

FIFA 11 was originally developed by the FIFA Medical Assessment and Research Centre (F-MARC) for adult football players, emphasizing running drills, dynamic stretching, and eccentric hamstring strengthening (Steffen et al., 2008).

FIFA 11+, the updated and evidence-based version, comprises 15 structured exercises divided into three phases: (1) dynamic running and movement preparation, (2) strength, balance, and plyometric training with controlled landing, and (3) football-specific running drills (Owoeye et al., 2014).

FIFA 11+ Kids adapts this model for younger players (7–13 years), incorporating playful coordination and balance games with progressive bodyweight strength training to promote engagement and motor control (Al Attar et al., 2023). Collectively, these programs aim to enhance neuromuscular control, optimize movement patterns, and reduce football-related injury risks across developmental stages.

Structured warm-up routines that incorporate strength and balance training have been associated with improved muscular endurance and joint stability, which may help reduce muscle strain injuries—as reflected by the pooled IRR of 0.62 in this meta-analysis.

However, the effectiveness of injury prevention programs varied depending on the content, implementation format, and fidelity of execution. According to the pooled findings of the present analysis, FIFA-based programs—particularly FIFA 11+ and FIFA 11+ Kids—demonstrated greater preventive efficacy than conventional neuromuscular training (NMT) protocols. Specifically, studies implementing FIFA 11+ or FIFA 11+ Kids and The 11 (Al Attar et al., 2023; Owoeye et al., 2014; Rössler et al., 2018; Soligard et al., 2008; Zarei et al., 2020) reported an average IRR of approximately 0.54, corresponding to a 46.0% reduction in overall injury incidence. In contrast, NMT-based interventions (Emery & Meeuwisse, 2010; Matias et al., 2021; Waldén et al., 2012) yielded weaker effects, with an average IRR of about 0.77, indicating a 23.0% reduction in injury risk. As some studies did not report overall injury data, knee injury outcomes—the most consistently reported endpoint—were used to estimate relative effectiveness. Several mechanisms may explain why FIFA 11+ Kids appears particularly effective in youth football players. From a biological and developmental perspective, FIFA 11+ Kids may offer advantages over traditional neuromuscular training (NMT) by integrating age-appropriate, football-specific movements with balance, coordination, and cognitive engagement. Unlike isolated NMT exercises, FIFA 11+ Kids emphasizes multi-planar actions, decision-making, and dynamic postural control within a sport-relevant context. These characteristics may better align with the neuromuscular and cognitive development of children, potentially enhancing movement quality and load tolerance during play and thereby reducing injury risk.

FIFA-based programs showed particularly strong effects in reducing lower limb (IRR ≈ 0.64), knee (IRR ≈ 0.59), and ankle injuries (IRR ≈ 0.68), whereas NMT protocols were more effective in preventing hip and groin injuries (IRR ≈ 0.44–0.55). The superior performance of FIFA 11+ and FIFA 11+ Kids may be attributed to their structured warm-up design, integration of dynamic balance and plyometric drills, and higher adherence rates, which collectively enhance neuromuscular control and transfer more effectively to real-game scenarios.

It is noteworthy that the preventive effect on non-contact injuries was relatively weaker (IRR = 0.73), consistent with previous studies (Lemes et al., 2021; Obertinca et al., 2023). This suggests that existing prevention programs may not fully address intrinsic risk factors such as fatigue-induced motor control errors, poor landing mechanics, or unstable gait patterns. Compared with contact injuries (IRR = 0.71), similar protective effects were observed for contact and non-contact injuries. One possible explanation is that injury prevention programs such as FIFA 11+ Kids may improve general movement quality, neuromuscular control, and postural stability, which could reduce injury risk across multiple mechanisms. Alternatively, the similarity in effect estimates may partly reflect limitations in injury mechanism classification, as contact and non-contact distinctions are not always clearly defined or reliably reported in field-based injury surveillance.

Substantial heterogeneity was observed for several outcomes, including overall injuries (*I*^2^ = 80.0%), lower-limb injuries (*I*^2^ = 72.3%), and contact injuries (*I*^2^ = 70.5%). Several factors may contribute to this variation. Compliance appears to be a critical determinant of program effectiveness. Beaudouin et al. (2019) reported a negative correlation between adherence to FIFA 11+ Kids and injury incidence among 3,895 child football players, indicating that higher compliance was associated with fewer injuries. Likewise, Steffen et al. (2013) found that high-adherence groups achieved significantly greater reductions in injury rates compared with low-adherence groups. However, most studies included in the present meta-analysis did not report compliance data in sufficient detail to enable quantitative synthesis, precluding a direct examination of adherence effects. This represents an important methodological limitation, as variability in program fidelity and implementation quality likely contributed to the observed heterogeneity. Future trials should systematically record and report adherence rates to identify the compliance thresholds required to achieve optimal preventive benefits.

Supervision and delivery mode also influence intervention efficacy. Sadigursky et al. (2017) observed that athletes receiving in-person guidance from professionals (*e.g.*, physical therapists or strength coaches) benefited more than those following online or video-based programs, underscoring the importance of expert supervision.

Participant characteristics—including age distribution, intervention duration, and training content—may further account for heterogeneity. Given the broad age range (3–18 years), physiological responses to identical training stimuli may vary across developmental stages (Granacher et al., 2016). Additionally, sex differences may also modulate intervention efficacy. Most included studies involved predominantly male participants, limiting the generalizability of findings to female football players. Previous research has shown that female athletes exhibit higher rates of knee and anterior cruciate ligament (ACL) injuries, likely due to differences in neuromuscular control, lower-limb alignment, and hormonal fluctuations across the menstrual cycle (Hewett, Myer & Ford, 2006; Sigward, Pollard & Powers, 2012). Consequently, prevention programs emphasizing landing mechanics, balance, and lower-limb strength may yield greater relative benefits for female athletes.

Because few included studies reported sex-stratified results, it was not possible to perform quantitative analyses by sex. Future research should incorporate sex-specific subgroup analyses to clarify whether male and female football players respond differently to structured injury prevention programs. Furthermore, intervention duration ranged from three months to one competitive season, which may have influenced the comparability of injury incidence trends.

Finally, variations in program content and design likely contributed to additional heterogeneity. For example, Alhazmi et al. (2025) directly compared FIFA 11 and FIFA 11+ in preventing ankle injuries and found that FIFA 11+ produced superior outcomes, suggesting that even similarly named programs may differ in training intensity and effectiveness. Although this study examined multiple injury types, it did not conduct direct head-to-head comparisons between programs due to limited sample sizes and high between-study heterogeneity. Future large-scale randomized trials should therefore evaluate the relative efficacy of specific injury prevention strategies for targeted injury types.

### Effectiveness of injury prevention programs across age groups

This study conducted an age-stratified subgroup analysis based on seven randomized controlled trials. The results showed that both the children’s group (IRR = 0.54) and the adolescent group (IRR = 0.68) benefited significantly from the injury prevention programs, with only a modest difference between the two. This small variation may reflect overlapping developmental characteristics and similarities in intervention content across studies—for instance, most trials adopted the FIFA 11 series of injury prevention programs, which share highly consistent structures and training components—rather than a true difference in the intrinsic efficacy of the interventions.

Additionally, the limited number of studies in each subgroup (a total of seven) and variations in intervention type, duration, and sample size may have led to overlapping confidence intervals, thereby reducing the statistical distinction between age groups. Despite the modest difference, the intervention effect in the children’s group remained slightly stronger than that in adolescents.

Further subgroup analyses revealed that the children’s group (<13 years) not only exhibited a greater overall effect size (IRR = 0.54) but also demonstrated lower heterogeneity, whereas the adolescent group (13–18 years) showed greater variability across studies. These discrepancies are likely associated with physiological and neuromuscular differences related to developmental stage. Prepubertal children generally possess more stable musculoskeletal structures and lower cumulative training loads, allowing for more uniform and sustained neuromuscular adaptations. In contrast, adolescents undergo rapid physiological and hormonal changes during the period of peak height velocity (PHV), which may transiently impair coordination, increase joint loading, and elevate injury susceptibility (Le Gall, Carling & Reilly, 2007; Pakarinen, Ponkilainen & Kuitunen, 2025; Wik, 2022). Such factors may also contribute to greater heterogeneity and less consistent intervention effects across adolescent populations.

The superior outcomes observed in children may also reflect differences in physiological maturation, motor skill plasticity, and training experience. Children are in a critical period of motor skill development, making them particularly responsive to interventions targeting postural control, dynamic balance, and core stability. Additionally, most children have limited prior training experience, and their initial exposure to structured, multicomponent prevention programs may elicit a “novelty effect,” resulting in pronounced short-term improvements (Behringer et al., 2011; Lopes et al., 2020; Tymofiyeva & Gaschler, 2020).

Conversely, adolescents experience rapid somatic growth, hormonal fluctuations, and increased training loads, which can challenge the stability of neuromuscular control and reduce responsiveness to preventive interventions. Moreover, adolescents often face higher training volumes, more frequent competition, and greater physical contact, making it more difficult for a single intervention to counterbalance the cumulative influence of external risk factors.

The relatively lower risk ratio observed among children may also reflect the absence of prior systematic prevention strategies. In typical training settings, child football players often lack specialized warm-up routines, strength programs, or injury prevention education. Therefore, introducing comprehensive, multicomponent interventions—including dynamic stretching, stability training, and neuromuscular control—can generate larger marginal benefits and produce more substantial reductions in injury risk.

Collectively, these findings underscore the importance of tailoring injury prevention programs to the physiological and training characteristics of different developmental stages, rather than employing a one-size-fits-all model. In particular, implementing structured prevention strategies early—during the sensitive period of motor skill development—may help establish a stronger foundation for long-term athletic health and performance sustainability in young football players.

### Comparison with previous studies

To date, meta-analyses focusing specifically on injury prevention programs for child and adolescent athletes remain limited. In a recent systematic review covering all age groups (children, adolescents, and adults), Obertinca et al. (2023) reported a pooled relative risk (RR) of 0.52 for overall injuries in children, closely aligning with the IRR of 0.54 observed in the present study for child and adolescent populations. However, the Obertinca review included a broad participant spectrum and exhibited considerable heterogeneity, with a limited number of primary studies focused specifically on children and adolescents. As a result, the stability and applicability of their findings for younger athletes may be limited. In contrast, the current meta-analysis is dedicated exclusively to child and adolescent football players, with more focused inclusion criteria, yielding findings that are both more representative and robust for this population.

In adult cohorts, Van Beijsterveldt et al. (2012) reported no significant reduction in injury incidence following implementation of the FIFA 11 program among adult football players. This finding supports the notion that injury prevention programs may exhibit greater efficacy and adaptability in younger populations than in adults. Similarly, Wik et al. (2021) examined the relationship between injury risk and age, demonstrating a significant increase in injury incidence with advancing age, which indirectly explains the more pronounced preventive benefits observed among children and adolescents in the present study.

Additional evidence from Rössler et al. (2018) demonstrated a 48.0% reduction in overall injury incidence in the intervention group compared with controls following implementation of the FIFA 11+ Kids program—findings that align closely with the results of the current meta-analysis. In contrast, Crossley et al. (2020) reported only a 27.0% reduction in overall injury incidence in a study focusing on adult female football players. Collectively, these comparisons suggest that, under similar intervention conditions, children and adolescents exhibit greater responsiveness to injury prevention programs than adult or female cohorts.

Similarly, Thorborg et al. (2017), in their analysis of FIFA-endorsed prevention protocols (FIFA 11 and FIFA 11+), reported an approximate 25.0% reduction in overall injury risk per 1000 h of exposure—considerably lower than the 42.0% risk reduction observed in children and adolescents in the present study. This further underscores the differential impact of injury prevention programs across distinct population groups.

Such differences may be attributed to variations in physiological development and training adaptation mechanisms. For example, male athletes may be more susceptible to acute, high-impact injuries due to greater contact intensity and movement demands, while female athletes are at higher risk for certain types of overuse injuries related to lower muscle strength and pelvic structure (Alahmad et al., 2024). Child and adolescent athletes, on the other hand, benefit from greater neuroplasticity and adaptability during the developmental stage, making them more responsive to structured training programs and yielding better preventive outcomes.

Notably, the effect size for lower limb injury reduction observed in this study exceeds that reported in previous studies involving adults. For example, Finch et al. (2016) observed only modest reductions in lower limb injuries among adult populations, whereas the current meta-analysis identified a much more pronounced protective effect in child and adolescent athletes. Collectively, these comparative findings highlight not only the greater effectiveness but also the higher practical value of implementing injury prevention programs in younger athletic populations.

### Strengths and limitations

This study presents several notable strengths. First, it provides a comprehensive and multidimensional evaluation of injury prevention programs among child and adolescent football players, covering overall, site-specific (knee, ankle, hip/groin, and muscle), and contact *vs.* non-contact injuries. This inclusive approach strengthens the assessment of program effectiveness in this population.

Second, the inclusion of age-stratified subgroup analyses enhances the precision and clinical relevance of the findings by comparing the intervention effects between children and adolescents. Third, this study expands previous work by systematically evaluating muscle injuries, an area often underrepresented in prior literature. For instance, Obertinca et al. (2023) focused solely on hamstring injuries, and Thorborg et al. (2017) did not assess muscle-related outcomes comprehensively. The inclusion of newer evidence (Al Attar et al., 2023; Matias et al., 2021) provides broader coverage and improves the credibility of conclusions. Furthermore, the large overall sample size (*n* = 16,636) offers sufficient statistical power and representativeness.

However, several limitations should be acknowledged. Considerable heterogeneity (I^2^ > 80.0%) was observed in outcomes such as overall, lower-limb, and contact injuries, likely reflecting differences in intervention duration, implementation, and compliance. The wide age range of participants (3–18 years) also introduces developmental variability, though this was partially mitigated through stratification based on WHO and IOC definitions (<13 years for children, 13–18 years for adolescents). Some overlap in developmental stages may remain.

Information size was limited for several secondary outcomes. As shown in Table S3, *post-hoc* power and MDES analyses suggest that some outcomes (*e.g.*, hip/groin, contact, and non-contact injuries) had power <0.80 at *α* = 0.05, indicating reduced sensitivity to detect modest but clinically meaningful effects. Although we performed *post-hoc* power and MDES analyses (Table S3), we did not conduct an a priori optimal information size (OIS) assessment or trial sequential analysis (TSA). The absence of OIS/TSA limits the ability to determine whether the accumulated evidence is sufficient to draw firm conclusions, particularly for outcomes with small numbers of studies or wide confidence intervals. Therefore, findings for underpowered outcomes should be interpreted with caution. Such approaches may further strengthen inference for the primary outcome; however, they were beyond the scope of the present review and require additional assumptions. Future updates could consider OIS/TSA to evaluate whether the accrued information size is sufficient for firm conclusions. Therefore, non-significant or borderline findings with wide 95.0% CIs should be interpreted as imprecise rather than definitive evidence of no effect. Future trials with larger samples/exposure and harmonised injury ascertainment are needed to improve precision.

Most studies used established injury surveillance approaches (*e.g.*, IRFs or web-based systems) aligned with consensus definitions (Fuller et al., 2006); however, formal reliability statistics (*e.g.*, kappa/ICC/test–retest) were rarely reported (Table S4), which may limit comparability across observers and sites. Several included trials used cluster randomization (*e.g.*, by team or school). Because most trials did not report ICCs or cluster-adjusted effect estimates, we were unable to adjust for clustering using effective sample size methods. Ignoring clustering may overestimate the precision of pooled estimates (*i.e.,* produce narrower confidence intervals). Therefore, results—particularly for secondary outcomes—should be interpreted cautiously.

In addition, only nine randomized controlled trials were included, and the number of studies for certain secondary outcomes (*e.g.*, hip/groin and muscle injuries) was limited, potentially reducing the power of subgroup analyses. Most trials also featured short follow-up periods (≤ one season), restricting conclusions on long-term effects.Most included trials implemented the intervention two to three times per week; however, dose–response relationships could not be formally examined in this review. Heterogeneous reporting of training frequency, session duration, and adherence precluded quantitative subgroup analyses. Consequently, it remains unclear whether higher intervention frequency confers additional protective benefits or whether a minimum effective dose exists.

The generalizability of results is further constrained by participant homogeneity: most studies focused on male players within similar age brackets, and few clearly described control warm-up protocols or adherence. Moreover, the predominance of studies from developed Western countries limits global applicability. Some potentially relevant studies were excluded due to differences in intervention content (Zebis et al., 2018), which may have omitted alternative preventive strategies. Additionally, most included studies were conducted in European countries, which may limit the generalizability of the findings to other football cultures and regions, such as South America, Africa, or Asia, where training structures and playing conditions may differ.

Moreover, sex-specific effects could not be adequately explored, as most included studies predominantly enrolled male players. Given established sex differences in anterior cruciate ligament (ACL) injury risk and neuromuscular control during adolescence, the effectiveness of FIFA 11+ Kids in female players warrants further investigation.

Finally, although publication bias was assessed using Egger’s and Begg’s tests, the exclusion of gray literature means residual bias cannot be ruled out. Future reviews should employ more inclusive search strategies and standardized reporting to enhance reproducibility and cross-context applicability. Additionally, this review was registered retrospectively in PROSPERO, which may limit the ability to independently verify that outcomes were not selectively modified after data analysis had begun, thereby introducing a potential risk of reporting bias. However, no deviations from the original study objectives were made during the review process.

### Practical implications and future research directions

Although numerous studies have confirmed the injury-preventive effects of programs such as FIFA 11, FIFA 11+, and FIFA 11+ Kids among child and adolescent football players, significant challenges remain regarding their practical implementation and long-term sustainability (Minnig et al., 2022). Among these, training compliance is recognized as a crucial determinant of intervention effectiveness

Franchina et al., (2023) and colleagues have pointed out that while the FIFA 11 series is empirically supported for injury prevention, the training routines are often mechanical and repetitive, lacking elements of fun. This can diminish motivation and engagement among young athletes, subsequently reducing actual compliance and the effectiveness of the intervention. Therefore, it is advisable in practice to moderate the frequency of such training routines, avoiding excessive repetition that could lead to reduced adherence.

Additionally, there is evidence that systematic education regarding injury risk and prevention awareness can further reduce the incidence of sports injuries among football players (Alahmad et al., 2021). Consequently, when designing interventions for adolescents and children, attention should be paid not only to the scientific rigor and effectiveness of the training itself but also to enhancing its enjoyment and interactivity. This can promote greater participant engagement and sustained adherence. It is also recommended to incorporate systematic injury prevention education throughout the intervention process, strengthening safety awareness and behavioral norms, thereby fostering intrinsic motivation for long-term safe participation.

Future research should further explore the following aspects:

Long-term follow-up and adherence monitoring: future trials should incorporate longer follow-up periods of at least two competitive seasons, with regular (*e.g.*, quarterly) monitoring of intervention adherence, to evaluate long-term effectiveness and potential declines in compliance over time.

Head-to-head randomized controlled trials: adequately powered head-to-head randomized controlled trials comparing FIFA 11+, FIFA 11+ Kids, and traditional neuromuscular training programs within comparable populations are needed to determine their relative effectiveness and inform age- and context-specific implementation strategies.

Dose–response relationships: dose–response effects should be examined using meta-regression approaches or individual participant data (IPD) meta-analyses to identify the minimal effective training dose and optimal intervention frequency for injury prevention in youth football.

Objective assessment of training load and movement quality: future studies may benefit from integrating objective assessment tools, such as wearable sensors (*e.g.*, accelerometers and GPS devices) and biomechanical analyses, to quantify training load, movement quality, and their interaction with injury prevention outcomes.

Refining age-stratified analyses: exploring the differential effects of interventions across developmental stages (*e.g.*, children *vs.* adolescents) can inform the optimization of individualized prevention strategies.

Expanding geographic scope: currently, most high-quality evidence is concentrated in Europe and North America. Future studies should include more research from Africa, Asia, and other developing regions to fill existing data gaps and improve the global representativeness of the evidence base.

From a practical perspective, it is recommended that injury prevention programs be routinely integrated into child and adolescent football clubs, schools, and community training systems. Implementation should be accompanied by guidance from sports medicine professionals or strength and conditioning coaches to enhance scientific rigor and execution quality. Furthermore, intervention content should be adapted to the preferences of young athletes, with the development of more interactive and engaging training formats to maximize both the enjoyment and sustained effectiveness of injury prevention initiatives.

## Conclusion

Based on nine randomized controlled trials, this meta-analysis systematically evaluated the effects of structured injury prevention programs—including FIFA 11+, FIFA 11+ Kids, and neuromuscular training—on injury risk among child and adolescent football players. The results demonstrate that these interventions significantly reduce the incidence of both overall and site-specific injuries, confirming their broad preventive benefits.

For overall injuries, the intervention group showed an approximately 38.0% lower injury risk compared with controls (IRR = 0.62, 95.0% CI [0.49–0.78]). For hip/groin injuries, the pooled estimate suggested a potential reduction (IRR = 0.51); however, this finding was not statistically significant (95.0% CI [0.24–1.09]) and was based on limited evidence (three trials). Therefore, it should be interpreted as suggestive but inconclusive. Significant risk reductions were also observed for knee (IRR = 0.67), lower limb (IRR = 0.68), and ankle injuries (IRR = 0.72). Reductions were observed for both contact (IRR = 0.71) and non-contact injuries (IRR = 0.73); however, these mechanism-specific analyses were based on fewer trials/participants and were underpowered, so the findings should be interpreted cautiously and considered exploratory.

Notably, all pooled incidence rate ratios (IRRs) were below 1, emphasizing the overall protective role of injury prevention programs in youth football. Nevertheless, the analysis was limited by moderate-to-high heterogeneity (*e.g.*, *I*^2^ = 80.0% for overall injuries). This could be attributed to the absence of reliability data (such as Kappa values and ICC) in several studies, particularly in multi-center studies like Rössler et al. (2018), presents a limitation in our ability to assess the consistency of injury classifications. This missing data introduces uncertainty and contributes to heterogeneity in the meta-analysis. Future studies should prioritize the inclusion of reliability measures to ensure the robustness of injury surveillance results. Small sample sizes for certain subgroups, incomplete compliance data, and limited long-term follow-up. These factors should be interpreted cautiously when generalizing the results.

Despite these limitations, the evidence strongly supports the regular integration of structured injury prevention programs into youth football training—particularly during the pre-season and early training phases. Implementing scientifically validated programs such as FIFA 11+ and neuromuscular training can effectively reduce short-term injury risk and lay the foundation for long-term physical resilience and performance development.

Future research should shift from broad validation to more targeted and feasible approaches. Comparative trials under standardized conditions are needed to identify the most effective components of FIFA 11+, FIFA 11+ Kids, and neuromuscular training programs for specific age groups. Studies should also examine how sex and maturational differences affect injury mechanisms and intervention responses. Moreover, participant compliance, supervision quality, and load management should be investigated as mediating factors influencing outcomes. Long-term prospective studies are warranted to evaluate whether these preventive effects persist across competitive seasons.

By addressing these gaps, future research can help translate current evidence into practical, evidence-based models that enhance both safety and sustainable athletic development among child and adolescent football players.

##  Supplemental Information

10.7717/peerj.21319/supp-1 Supplemental Information 1 PRISMA checklist

10.7717/peerj.21319/supp-2 Supplemental Information 2 Raw data Data from Review Manager (RevMan) and Stata.

10.7717/peerj.21319/supp-3 Supplemental Information 3 Supplementary Materials The additional materials supporting the main article, including full search strategies, data extraction form, *post-hoc* power and minimal detectable effect size analyses, injury assessment and measurement specifications, and supplementary figures such as funnel plots and sensitivity analyses.

## Additional Information and Declarations

### Data Availability

The following information was supplied regarding data availability: The data is available in the Supplementary File. The code is available at Zenodo: HongyuYang. (2026). Data and Stata code for meta-analysis (overall and subgroup analyses) [Data set]. Zenodo. https://doi.org/10.5281/zenodo.19554138.

## References

[ref-1] Al Attar WSA, Bizzini M, Alzahrani H, Alarifi S, Ghulam H, Alyami M, Alzhrani M, Sanders RH (2023). The FIFA 11+ kids injury prevention program reduces injury rates among male children soccer players: a clustered randomized controlled trial. Sports Health.

[ref-2] Alahmad TA, Tierney AC, Boland P, Clifford AM (2024). Injury risk and prevention strategies among Saudi and Irish amateur women soccer players—a qualitative study. International Journal of Physical Therapy Research & Practice.

[ref-3] Alahmad TA, Tierney AC, Cahalan RM, Almaflehi NS, Clifford AM (2021). Injury risk profile of amateur Irish women soccer players and players’ opinions on risk factors and prevention strategies. Physical Therapy in Sport.

[ref-4] Alhazmi M, Alhazmi E, Alghamdi WA, Zalah M, Uddin S, Rizvi MR, Ahmad F (2025). Effectiveness of FIFA injury prevention programs in reducing ankle injuries among football players: a systematic review. PeerJ.

[ref-5] Beaudouin F, Rossler R, Funten KAD, Bizzini M, Chomiak J, Verhagen E, Junge A, Dvorak J, Lichtenstein E, Meyer T, Faude O (2019). Effects of the ‘11+Kids’ injury prevention programme on severe injuries in children’s football: a secondary analysis of data from a multicentre cluster-randomised controlled trial. British Journal of Sports Medicine.

[ref-6] Behringer M, Vom Heede A, Matthews M, Mester J (2011). Effects of strength training on motor performance skills in children and adolescents: a meta-analysis. Pediatric Exercise Science.

[ref-7] Bergeron MF, Mountjoy M, Armstrong N, Chia M, Côté J, Emery CA, Faigenbaum A, Hall Jr G, Kriemler S, Léglise M, Malina RM, Pensgaard AM, Sanchez A, Soligard T, Sundgot-Borgen J, Van Mechelen W, Weissensteiner JR, Engebretsen L (2015). International Olympic Committee consensus statement on youth athletic development. British Journal of Sports Medicine.

[ref-8] Bult HJ, Barendrecht M, Tak IJR (2018). Injury risk and injury burden are related to age group and peak height velocity among talented male youth soccer players. Orthopaedic Journal of Sports Medicine.

[ref-9] Crossley KM, Patterson BE, Culvenor AG, Bruder AM, Mosler AB, Mentiplay BF (2020). Making football safer for women: a systematic review and meta-analysis of injury prevention programmes in 11,773 female football (soccer) players. British Journal of Sports Medicine.

[ref-10] Emery CA, Van den Berg C, Richmond SA, Palacios-Derflingher L, McKay CD, Doyle-Baker PK, McKinlay M, Toomey CM, Nettel-Aguirre A, Verhagen E, Belton K, Macpherson A, Hagel BE (2020). Implementing a junior high school-based programme to reduce sports injuries through neuromuscular training (iSPRINT): a cluster randomised controlled trial (RCT). British Journal of Sports Medicine.

[ref-11] Emery CA, Meeuwisse WH (2010). The effectiveness of a neuromuscular prevention strategy to reduce injuries in youth soccer: a cluster-randomised controlled trial. British Journal of Sports Medicine.

[ref-12] Emery CA, Meeuwisse WH, Hartmann SE (2005). Evaluation of risk factors for injury in adolescent soccer: implementation and validation of an injury surveillance system. The American Journal of Sports Medicine.

[ref-13] Faude O, Rößler R, Junge A (2013). Football injuries in children and adolescent players: are there clues for prevention?. Sports Medicine.

[ref-14] Finch CF, Twomey DM, Fortington LV, Doyle TLA, Elliott BC, Akram M, Lloyd DG (2016). Preventing Australian football injuries with a targeted neuromuscular control exercise programme: comparative injury rates from a training intervention delivered in a clustered randomised controlled trial. Injury Prevention.

[ref-15] Franchina M, Turati M, Tercier S, Kwiatkowski B (2023). FIFA 11+ Kids: challenges in implementing a prevention program. Journal of Children’s Orthopaedics.

[ref-16] Fuller CW, Ekstrand J, Junge A, Andersen TE, Bahr R, Dvorak J, Hägglund M, McCrory P, Meeuwisse WH (2006). Consensus statement on injury definitions and data collection procedures in studies of football (soccer) injuries. British Journal of Sports Medicine.

[ref-17] Granacher U, Lesinski M, Busch D, Muehlbauer T, Prieske O, Puta C, Behm DG (2016). Effects of resistance training in youth athletes on muscular fitness and athletic performance: a conceptual model for long-term athlete development. Frontiers in Physiology.

[ref-18] Hewett TE, Myer GD, Ford KR (2006). Anterior cruciate ligament injuries in female athletes: part 1, mechanisms and risk factors. The American Journal of Sports Medicine.

[ref-19] Higgins JPT, Altman DG, Gotzsche PC, Juni P, Moher D, Oxman AD, Savovic J, Schulz KF, Weeks L, Sterne JAC (2011). The Cochrane Collaboration’s tool for assessing risk of bias in randomised trials. BMJ.

[ref-20] Hilska M, Leppaenen M, Vasankari T, Aaltonen S, Kannus P, Parkkari J, Steffen K, Kujala UM, Konttinen N, Raeisaenen AM, Pasanen K (2021). Neuromuscular training warm-up prevents acute noncontact lower extremity injuries in children’s soccer: a cluster randomized controlled trial. Orthopaedic Journal of Sports Medicine.

[ref-21] Le Gall F, Carling C, Reilly T (2007). Biological maturity and injury in elite youth football. Scandinavian Journal of Medicine & Science in Sports.

[ref-22] Lemes IR, Pinto RZ, Lage VN, Rocha BAB, Verhagen E, Bolling C, Aquino CF, Fonseca ST, Souza TR (2021). Do exercise-based prevention programmes reduce non-contact musculoskeletal injuries in football (soccer)? A systematic review and meta-analysis with 13,355 athletes and more than 1 million exposure hours. British Journal of Sports Medicine.

[ref-23] Lopes L, Santos R, Coelho E Silva M, Draper C, Mota J, Jidovtseff B, Clark C, Schmidt M, Morgan P, Duncan M, O’Brien W, Bentsen P, D’Hondt E, Houwen S, Stratton G, De Martelaer K, Scheuer C, Herrmann C, García-Hermoso A, Ramírez-Vélez R, Palmeira A, Gerlach E, Rosário R, Issartel J, Esteban-Cornejo I, Ruiz J, Veldman S, Zhang Z, Colella D, Póvoas S, Haibach-Beach P, Pereira J, McGrane B, Saraiva J, Temple V, Silva P, Sigmund E, Sousa-Sá E, Adamakis M, Moreira C, Utesch T, True L, Cheung P, Carcamo-Oyarzun J, Charitou S, Chillón P, Robazza C, Silva A, Silva D, Lima R, Mourão-Carvalhal I, Khodaverdi Z, Zequinão M, Pereira B, Prista A, Agostinis-Sobrinho C (2020). A narrative review of motor competence in children and adolescents: what we know and what we need to find out. International Journal of Environmental Research and Public Health.

[ref-24] López-Valenciano A, Ruiz-Pérez I, Garcia-Gómez A, Vera-Garcia FJ, DeSte Croix M, Myer GD, Ayala F (2020). Epidemiology of injuries in professional football: a systematic review and meta-analysis. British Journal of Sports Medicine.

[ref-25] Matias H, Mari L, Tommi V, Benjamin C, Sari A, Roald B, Heidi H, Jari P, Pekka K, Kati P (2021). Neuromuscular training warm-up has no effect on the prevalence of overuse lower extremity injuries in children’s soccer. A cluster randomized controlled trial Conference Abstract. Clinical Journal of Sport Medicine.

[ref-26] Minnig MC, Hawkinson L, Root HJ, Driban J, Di Stefano LJ, Callahan L, Ambrose KR, Spang JT, Golightly YM (2022). Barriers and facilitators to the adoption and implementation of evidence-based injury prevention training programmes: a narrative review. BMJ Open Sport & Exercise Medicine.

[ref-27] Obertinca R, Hoxha I, Meha R, Lama A, Bimbashi A, Kuqi D, Aus der Fuenten K, Shabani B, Meyer T (2023). Efficacy of multi-component exercise-based injury prevention programs on injury risk among footballers of all age groups: a systematic review and meta-analysis [review]. Sports Medicine.

[ref-28] Owoeye OBA, Akinbo SRA, Tella BA, Olawale OA (2014). Efficacy of the FIFA 11+warm-up programme in male youth football: a cluster randomised controlled trial. Journal of Sports Science and Medicine.

[ref-29] Page MJ, McKenzie JE, Bossuyt PM, Boutron I, Hoffmann TC, Mulrow CD, Shamseer L, Tetzlaff JM, Akl EA, Brennan SE, Chou R, Glanville J, Grimshaw JM, Hróbjartsson A, Lalu MM, Li T, Loder EW, Mayo-Wilson E, McDonald S, McGuinness LA, Stewart LA, Thomas J, Tricco AC, Welch VA, Whiting P, Moher D (2021). The PRISMA 2020 statement: an updated guideline for reporting systematic reviews. BMJ.

[ref-30] Pakarinen O, Ponkilainen VT, Kuitunen I (2025). Association of peak height velocity and skeletal maturity to injury incidence in male elite adolescent football (soccer) players-a systematic review. Health Science Reports.

[ref-31] Parry GN, Williams S, McKay CD, Johnson DJ, Bergeron MF, Cumming SP (2024). Associations between growth, maturation and injury in youth athletes engaged in elite pathways: a scoping review. British Journal of Sports Medicine.

[ref-32] Roessler R, Donath L, Verhagen E, Junge A, Schweizer T, Faude O (2014). Exercise-based injury prevention in child and adolescent sport: a systematic review and meta-analysis [review]. Sports Medicine.

[ref-33] Rössler R, Junge A, Bizzini M, Verhagen E, Chomiak J, aus der Fünten K, Meyer T, Dvorak J, Lichtenstein E, Beaudouin F, Faude O (2018). A multinational cluster randomised controlled trial to assess the efficacy of ‘11+ kids’: a warm-up programme to prevent injuries in children’s football. Sports Medicine.

[ref-34] Rössler R, Junge A, Chomiak J, Dvorak J, Faude O (2015). Soccer injuries in players aged 7 to 12 years. The American Journal of Sports Medicine.

[ref-35] Sadigursky D, Braid JA, Lemos De Lira DN, Barreto Machado BA, Fernandes Carneiro RJ, Colavolpe PO (2017). The FIFA 11+ injury prevention program for soccer players: a systematic review [Review]. BMC Sports Science Medicine and Rehabilitation.

[ref-36] Sigward SM, Pollard CD, Powers CM (2012). The influence of sex and maturation on landing biomechanics: implications for anterior cruciate ligament injury. Scandinavian Journal of Medicine & Science in Sports.

[ref-37] Soligard T, Myklebust G, Steffen K, Holme I, Silvers H, Bizzini M, Junge A, Dvorak J, Bahr R, Andersen TE (2008). Comprehensive warm-up programme to prevent injuries in young female footballers: cluster randomised controlled trial. BMJ.

[ref-38] Soomro N, Sanders R, Hackett D, Hubka T, Ebrahimi S, Freeston J, Cobley S (2016). The efficacy of injury prevention programs in adolescent team sports: a meta-analysis. The American Journal of Sports Medicine.

[ref-39] Steffen K, Meeuwisse WH, Romiti M, Kang J, McKay C, Bizzini M, Dvorak J, Finch C, Myklebust G, Emery CA (2013). Evaluation of how different implementation strategies of an injury prevention programme (FIFA 11+) impact team adherence and injury risk in Canadian female youth football players: a cluster-randomised trial. British Journal of Sports Medicine.

[ref-40] Steffen K, Myklebust G, Olsen OE, Holme I, Bahr R (2008). Preventing injuries in female youth football–a cluster-randomized controlled trial. Scandinavian Journal of Medicine & Science in Sports.

[ref-41] Su W, Wang J, Ying Y, Lu B, Liu H, Zhou Z, Liu C, Yun H (2025). Injury risk reduction programs including balance training reduce the incidence of anterior cruciate ligament injuries in soccer players: a systematic review and meta-analysis. Journal of Orthopaedic Surgery and Research.

[ref-42] Thorborg K, Krommes KK, Esteve E, Clausen MB, Bartels EM, Rathleff MS (2017). Effect of specific exercise-based football injury prevention programmes on the overall injury rate in football: a systematic review and meta-analysis of the FIFA 11 and 11+programmes [Review]. British Journal of Sports Medicine.

[ref-43] Tymofiyeva O, Gaschler R (2020). Training-induced neural plasticity in youth: a systematic review of structural and functional MRI studies. Frontiers in Human Neuroscience.

[ref-44] Valentin S, Linton L, Sculthorpe NF (2024). Effect of supervision and athlete age and sex on exercise-based injury prevention programme effectiveness in sport: a meta-analysis of 44 studies. Research in Sports Medicine.

[ref-45] Van Beijsterveldt AMC, Van de Port IGL, Krist MR, Schmikli SL, Stubbe JH, Frederiks JE, Backx FJG (2012). Effectiveness of an injury prevention programme for adult male amateur soccer players: a cluster-randomised controlled trial. British Journal of Sports Medicine.

[ref-46] Waldén M, Atroshi I, Magnusson H, Wagner P, Hägglund M (2012). Prevention of acute knee injuries in adolescent female football players: cluster randomised controlled trial. BMJ.

[ref-47] World Health Organization (2022). Adolescent health.

[ref-48] Wik EH (2022). Growth, maturation and injuries in high-level youth football (soccer): a mini review. Frontiers in Sports and Active Living.

[ref-49] Wik EH, Lolli L, Chamari K, Materne O, Di Salvo V, Gregson W, Bahr R (2021). Injury patterns differ with age in male youth football: a four-season prospective study of 1,111 time-loss injuries in an elite national academy. British Journal of Sports Medicine.

[ref-50] Zarei M, Abbasi H, Namazi P, Asgari M, Rommers N, Roessler R (2020). The 11+Kids warm-up programme to prevent injuries in young Iranian male high-level football (soccer) players: a cluster-randomised controlled trial. Journal of Science and Medicine in Sport.

[ref-51] Zebis MK, Thorborg K, Andersen LL, Moller M, Christensen KB, Clausen MB, Holmich P, Wedderkopp N, Andersen TB, Krustrup P (2018). Effects of a lighter, smaller football on acute match injuries in adolescent female football: a pilot cluster-randomized controlled trial. Journal of Sports Medicine and Physical Fitness.

