# Supplementary material for: Effectiveness of sports injury prevention programs for adolescent and children football players: a meta-analysis and systematic review
Source: PeerJ. 2026 Jun 1;14:e21319. doi: 10.7717/peerj.21319 (PMC13235686; doi:10.7717/peerj.21319)
Supplement: Supplemental Information 3 — The additional materials supporting the main article, including full search strategies, data extraction form, post-hoc power and minimal detectable effect size analyses, injury assessment and measurement specifications, and supplementary figures such as funnel plots and sensitivity analyses. [file peerj-14-21319-s003.docx]

# Supplementary Material S1. Full Search Strategies

Databases: PubMed, Cochrane Library, Embase, EBSCOhost, Web of Science Core Collection.

## PubMed

1=(soccer[MeSH Terms] OR football[Title/Abstract] OR Football European[Title/Abstract] OR European Football[Title/Abstract])

2= (Athlete[MeSH Terms] OR sport[Title/Abstract] OR player[Title/Abstract] OR players[Title/Abstract])

3= (Adolescents[Title/Abstract] OR Adolescence[Title/Abstract] OR Youth[Title/Abstract] OR Youths[Title/Abstract] OR Teens[Title/Abstract] OR Teen[Title/Abstract] OR Teenagers[Title/Abstract] OR Teenager[Title/Abstract])

4= (Prevent[MeSH Terms] OR prevention[Title/Abstract] OR preventing[Title/Abstract] OR injury[Title/Abstract] OR injuries[Title/Abstract] OR risk reduction[Title/Abstract] OR program[Title/Abstract] OR programme[Title/Abstract] OR intervention[Title/Abstract])

5=(neuromuscular[MeSH Terms] OR exercise[Title/Abstract] OR safe[Title/Abstract] OR prophylactic[Title/Abstract] OR FIFA[Title/Abstract] OR balance training[Title/Abstract] OR strength[Title/Abstract] OR proprioceptive[Title/Abstract] OR movement training[Title/Abstract] OR conditioning[Title/Abstract] OR warm-up[Title/Abstract] OR warm up[Title/Abstract] OR therapy[Title/Abstract] OR load[Title/Abstract] OR nutrition[Title/Abstract] OR diet[Title/Abstract] OR sleep[Title/Abstract] OR educate[Title/Abstract] OR education[Title/Abstract] OR advice[Title/Abstract] OR strategy[Title/Abstract] OR strategies[Title/Abstract] OR wellness[Title/Abstract] OR psychological[Title/Abstract] OR cognitive[Title/Abstract] OR bracing[Title/Abstract] OR strapping[Title/Abstract] OR taping[Title/Abstract])

6=(Injury rate[MeSH Terms] OR injury risk[Title/Abstract] OR incidence[Title/Abstract] OR burden[Title/Abstract] OR prevalence[Title/Abstract])

7=(Randomised controlled trials[MeSH Terms] OR randomized controlled trials[Title/Abstract] OR RCT[Title/Abstract] OR randomized[Title/Abstract] OR randomised[Title/Abstract] OR controlled trials[Title/Abstract])

Last searched: [July 10 Month2026]

## Cochrane Library

1="Warm-Up Exercise":ti,ab,kw or "Warmup Exercise":ti,ab,kw or "Warm-Up Exercises":ti,ab,kw or "Warmup Exercises":ti,ab,kw or "Exercise, Warm-Up":ti,ab,kw or "Exercises, Warmup":ti,ab,kw or "Warming-Up Exercises":ti,ab,kw or "Exercise, Warming-Up":ti,ab,kw or "Exercises, Warm-Up":ti,ab,kw or "Warming Up Exercise":ti,ab,kw or "Exercise, Warmup":ti,ab,kw or "Warming-Up Exercise":ti,ab,kw or "Exercises, Warming-Up":ti,ab,kw or "Warm Up Exercise":ti,ab,kw
2="Injury, Sports":ti,ab,kw or "Injuries, Athletic":ti,ab,kw or "Injuries, Sports":ti,ab,kw or "Sports Injuries":ti,ab,kw or "Sports Injury":ti,ab,kw or "Athletic Injury":ti,ab,kw or "Injury, Athletic":ti,ab,kw
3="Primordial Preventions":ti,ab,kw or "Primordial Prevention":ti,ab,kw or "Prevention, Primordial":ti,ab,kw or "Preventions, Primordial":ti,ab,kw or "Disease Preventions":ti,ab,kw or "Primary Disease Prevention":ti,ab,kw or "Primary Disease Preventions":ti,ab,kw or "Prevention, Primary":ti,ab,kw or "Disease Prevention, Primary":ti,ab,kw
4="football":ti,ab,kw or "soccer":ti,ab,kw or "Football European":ti,ab,kw or "European, Football":ti,ab,kw or "Europeans, Football":ti,ab,kw or "European Football":ti,ab,kw
5="Adolescent":ti,ab,kw or "Adolescents":ti,ab,kw or "Youth":ti,ab,kw or "Adolescence":ti,ab,kw or "Youths":ti,ab,kw or "Teens":ti,ab,kw or "Teen":ti,ab,kw or "Teenagers":ti,ab,kw or "Teenager":ti,ab,kw
6="Athlete":ti,ab,kw or "sport":ti,ab,kw or "player":ti,ab,kw or "players":ti,ab,kw o
7="Prevent":ti,ab,kw or "preventing":ti,ab,kw or "prevention":ti,ab,kw or "injury":ti,ab,kw or "injuries":ti,ab,kw or "risk reduction":ti,ab,kw or "program":ti,ab,kw or "programme":ti,ab,kw or "intervention":ti,ab,kw
8="neuromuscular":ti,ab,kw or "exercise":ti,ab,kw or "safe":ti,ab,kw or "prophylactic":ti,ab,kw or "FIFA":ti,ab,kw or "balance training":ti,ab,kw or "strength":ti,ab,kw or "proprioceptive":ti,ab,kw or "movement training":ti,ab,kw or "warm-up":ti,ab,kw or "conditioning":ti,ab,kw or "therapy":ti,ab,kw or "warm up":ti,ab,kw or "load":ti,ab,kw or "nutrition":ti,ab,kw or "diet":ti,ab,kw or "educate":ti,ab,kw or "education":ti,ab,kw or "advice":ti,ab,kw or "strategies":ti,ab,kw or "wellness":ti,ab,kw or "psychological":ti,ab,kw or "cognitive":ti,ab,kw or "bracing":ti,ab,kw or "strapping":ti,ab,kw or "taping":ti,ab,kw or "sleep":ti,ab,kw or "strategy":ti,ab,kw

Last searched: 10 July 2026

## Embase

1='Primordial Preventions':ab,ti or 'Prevention, Primordial':ab,ti or 'Primordial Prevention':ab,ti or 'Preventions, Primordial':ab,ti or 'Disease Preventions':ab,ti or 'Primary Disease Prevention':ab,ti or 'Prevention, Primary':ab,ti or 'Primary Disease Preventions':ab,ti or 'Disease Prevention, Primary':ab,ti
2='Injury, Sports':ab,ti or 'Injuries, Athletic':ab,ti or 'Injuries, Sports':ab,ti or 'Sports Injuries':ab,ti or 'Sports Injury':ab,ti or 'Athletic Injury':ab,ti or 'Injury, Athletic':ab,ti
3='Adolescents':ab,ti or 'Adolescence':ab,ti or 'Youths':ab,ti or 'Youth':ab,ti or 'Teens':ab,ti or 'Teen':ab,ti or 'Teenagers':ab,ti or 'Teenager':ab,ti
4='randomized':ab,ti or 'randomized controlled trial':ab,ti or 'placebo':ab,ti
'Football European':ab,ti or 'European, Football':ab,ti or 'Europeans, Football':ab,ti or 'European Football':ab,ti or 'soccer':ab,ti
5='sport':ab,ti or 'Athlete':ab,ti or 'players':ab,ti or 'player':ab,ti
6='prevention':ab,ti or 'Prevent':ab,ti or 'preventing':ab,ti or 'injury':ab,ti or 'injuries':ab,ti or 'risk reduction':ab,ti or 'programme':ab,ti or 'program':ab,ti or 'intervention':ab,ti
7='neuromuscular':ab,ti or 'exercise':ab,ti or 'safe':ab,ti or 'prophylactic':ab,ti or 'FIFA':ab,ti or 'balance training':ab,ti or 'strength':ab,ti or 'proprioceptive':ab,ti or 'movement training':ab,ti or 'conditioning':ab,ti or 'warm-up':ab,ti or 'warm up':ab,ti or 'load':ab,ti or 'therapy':ab,ti or 'nutrition':ab,ti or 'diet':ab,ti or 'educate':ab,ti or 'advice':ab,ti or 'education':ab,ti or 'strategies':ab,ti or 'psychological':ab,ti or 'wellness':ab,ti or 'cognitive':ab,ti or 'bracing':ab,ti or 'strapping':ab,ti or 'taping':ab,ti or 'sleep':ab,ti or 'strategy':ab,ti

Last searched: 10 July 2026

## EBSCOhost

1=AB "Primordial Preventions" OR AB "Prevention, Primordial" OR AB "Primordial Prevention" OR AB "Preventions, Primordial" OR AB "Disease Preventions" OR AB "Primary Disease Prevention" OR AB "Prevention, Primary" OR AB "Primary Disease Preventions" OR AB "Disease Prevention, Primary"
2=AB "Injury, Sports" OR AB "Injuries, Athletic" OR AB "Injuries, Sports" OR AB "Sports Injuries" OR AB "Sports Injury" OR AB "Athletic Injury" OR AB "Injury, Athletic"
3=AB "Warm-Up Exercise" OR AB "Warm-Up Exercises" OR AB "Warmup Exercise" OR AB "Warmup Exercises" OR AB "Exercise, Warm-Up" OR AB "Warming-Up Exercises" OR AB "Exercises, Warmup" OR AB "Exercise, Warming-Up" OR AB "Exercises, Warm-Up" OR AB "Warming Up Exercise" OR AB "Exercise, Warmup" OR AB "Warming-Up Exercise" OR AB "Exercises, Warming-Up" OR AB "Warm Up Exercise"
4=AB Adolescents OR AB Adolescence OR AB Youth OR AB Youths OR AB Teens OR AB Teen OR AB Teenagers OR AB Teenager
5=AB randomized OR AB "randomized controlled trial" OR AB placebo
6=AB "Football European" OR AB "European, Football" OR AB "Europeans, Football" OR AB "European Football" OR AB soccer
7=AB Athlete OR AB sport OR AB player OR AB players
AB Prevent OR AB preventing OR AB prevention OR AB injury OR AB injuries OR AB "risk reduction" OR AB program OR AB intervention OR AB programme
8=AB neuromuscular OR AB exercise OR AB safe OR AB prophylactic OR AB FIFA OR AB strength OR 9=AB "balance training" OR AB proprioceptive OR AB "movement training" OR AB conditioning OR AB "warm-up" OR AB "warm up" OR AB therapy OR AB load OR AB nutrition OR AB diet OR AB education OR AB educate OR AB strategies OR AB advice OR AB wellness OR AB psychological OR 10=AB cognitive OR AB bracing OR AB strapping OR AB sleep OR AB taping OR AB strategy
11=AB "Injury rate" OR AB "injury risk" OR AB incidence OR AB burden OR AB prevalence

Last searched: 10 July 2026

## Web of Science Core Collection

#1TS=(Athlete OR sport OR player OR player)

#2TS=(Adolescents OR Adolescence OR Youth OR Youths OR Teens OR Teen OR Teenagers OR children)

#3TS=(Prevent OR prevention OR preventing OR injury OR injuries OR risk reduction OR program OR programme OR intervention)

#4TS=(neuromuscular OR exercise OR safe OR prophylactic OR FIFA OR balance training OR strength OR proprioceptive OR movement training OR conditioning OR warm-up OR warm up OR therapy OR load OR nutrition OR diet OR sleep OR educate OR education OR advice OR strategy OR strategies OR wellness OR psychological OR cognitive OR bracing OR strapping OR taping)

#5TS=(Injury rate OR injury risk OR incidence OR burden OR prevalence)

#6TS=(Randomised controlled trials OR randomized controlled trials OR RCT OR randomized OR randomised OR controlled trials)

#7TS=(soccer OR football OR Football European OR European Football)

Last searched: 10 July 2026

# Supplementary **Table S2**. Data Extraction Form

| Study ID | Country | Study design | Participants (n, age, sex) | Intervention (content, duration, frequency) | Comparator | Injury definition | Outcomes assessed | Exposure time | Effect estimates (IRR/RR/OR) | Follow-up period | Source (main text / supplement) | Notes |
| --- | --- | --- | --- | --- | --- | --- | --- | --- | --- | --- | --- | --- |

**Supplementary Table S3. Post-hoc power and minimal detectable effect size (MDES)**

Two-sided alpha=0.05. SE of ln(IRR) derived from 95% CI: SE = [ln(Upper)-ln(Lower)]/(2*1.96). MDES computed at 80% power.

| **Outcome** | **k (studies)** | **Pooled IRR** | **95% CI (L-U)** | **SE of ln(IRR)** | **Post-hoc power (alpha=0.05)** | **MDES @80% power (minimal detectable IRR <=)** | **Interpretation** |
| --- | --- | --- | --- | --- | --- | --- | --- |
| Overall injuries | 7 | 0.62 | 0.49-0.78 | 0.1186 | 0.98 | 0.72 | Adequately powered |
| Lower limb injuries | 8 | 0.68 | 0.56-0.82 | 0.0973 | 0.98 | 0.76 | Adequately powered |
| Knee injuries | 9 | 0.69 | 0.58-0.83 | 0.0914 | 0.98 | 0.77 | Adequately powered |
| Ankle injuries | 8 | 0.72 | 0.61-0.86 | 0.0876 | 0.96 | 0.78 | Adequately powered |
| Muscle injuries | 3 | 0.62 | 0.45-0.84 | 0.1592 | 0.85 | 0.64 | Adequately powered |
| Hip/groin injuries | 3 | 0.51 | 0.29-0.87 | 0.2803 | 0.67 | 0.46 | Underpowered (<0.80) |
| Contact injuries | 5 | 0.71 | 0.55-0.93 | 0.1340 | 0.72 | 0.69 | Underpowered (<0.80) |
| Non-contact injuries | 5 | 0.73 | 0.54-0.99 | 0.1546 | 0.53 | 0.65 | Underpowered (<0.80) |
| Children subgroup (overall) | 7 | 0.54 | 0.41-0.70 | 0.1365 | 0.99 | 0.68 | Adequately powered |
| Adolescents subgroup (overall) | 7 | 0.68 | 0.50-0.94 | 0.1610 | 0.67 | 0.64 | Underpowered (<0.80) |

**Supplementary Table S4. Injury assessment and measurement specifications (template)**

| **Study (Author, Year)** | **Outcome(s) assessed (overall/site-specific/contact/non-contact)** | **Injury definition (time-loss/medical attention/any complaint)** | **Classification system / criteria (name + key reference)** | **Surveillance / recording method (how injuries were recorded)** | **Who assessed / diagnosed? (physician/physio/AT/coach/self-report)** | **Assessment frequency & follow-up** | **Validity / ref(s) (injury definition/criteria)** | **Reliability / agreement (kappa/ICC/etc.)** | **Notes (NR/unclear)** |
| --- | --- | --- | --- | --- | --- | --- | --- | --- | --- |
| (Steffen 2008) | Overall injuries; site-specific (e.g., lower limb, knee, ankle); contact vs non-contact; acute vs overuse; recurrent; severity (minor/moderate/major). | Time-loss injury: registered if player was unable to fully take part in the next match or training session. | Consensus injury definitions/data collection procedures (Fuller et al., 2006). Severity by absence: minor (1–7 d), moderate (8–21 d), major (>21 d). Acute vs overuse and recurrent injury definitions applied. | All injuries recorded using a web-based recording system. Injured players interviewed by injury recorders using a standardized injury questionnaire. Coaches contacted by telephone/e-mail at least monthly to record training/match activity and new injuries. | 18 physical therapists served as injury recorders; blinded to group allocation. Moderate/major injuries typically diagnosed at a medical center (clinical tests/imaging/surgery as needed); minor injuries assessed by local physio/coach or not at all. | Prospective season surveillance: Mar 1–Oct 31, 2005; coach contacts at least monthly; injuries recorded throughout. | Fuller et al., 2006; van Mechelen et al., 1992. Registration method reliability/validity discussed previously and found adequate. (Olsen et al. 2006) | NR (no kappa/ICC/test–retest statistics reported). | NR |
| (AlAttar 2023) | Overall injury, lower extremity injuries, contact/non-contact, recurrent injuries, injury severity. | Injury: physical complaint preventing participation in subsequent training/match. | Classified by region (lower limb), mechanism (contact/non-contact), severity (mild, moderate, severe). | Weekly injury data recorded via Sports Injury Tracker by medical staff/coaches. | Medical staff (physiotherapists) and coaches; physicians for severe injuries. | 6 months (Aug 2018–Jan 2019); weekly injury data collection. | Based on Australian Sports Injury Data Dictionary. | NR (no kappa/ICC/test–retest statistics reported). | NR |
| (Emery  2010) | Overall injury rate, acute injury, lower-extremity injury, ankle sprain, knee sprain. | Soccer injury resulting in medical attention, removal from session, and/or time-loss from participation. | Injury classified by mechanism (acute vs overuse), body region (lower extremity), and severity (mild, moderate, severe) . | Injury surveillance using injury report forms (IRFs), baseline medical questionnaires, weekly exposure data, and follow-up by study therapist . | Study therapists (physiotherapists or athletic therapists) conducted injury assessments on-site. Injuries were recorded and categorized; follow-up conducted by the research coordinator. | Weekly exposure and injury data collected during the 20-week indoor soccer season (Oct 2006–Mar 2007). Follow-up continued until October 2007 for unresolved injuries. | IRFs previously validated in adolescent soccer populations(Emery et al. 2005) . | NR (no kappa/ICC/test–retest statistics reported). | No additional technical details on IRFs provided. |
| (Hilska 2021) | Acute lower extremity (LE) injuries, non-contact LE injuries, joint/ligament injuries, ankle injuries. | Any physical complaint sustained during soccer training or playing that resulted in injury, irrespective of medical attention or time-loss from participation. | Full injury definition as per Fuller et al., 2006. Injury severity classified by time-loss: slight (0 days), minor (1-3 days), mild (4-7 days), moderate (8-28 days), severe (>28 days). | Weekly injury data collected via SMS from parents, followed by structured interviews with injured players or their guardians. Data collection conducted by four blinded physical therapists. | njuries assessed by study physical therapists; follow-up and diagnosis were confirmed through interviews. | 20-week intervention with weekly injury reporting; follow-up until full return to play. | Based on consensus injury definitions by Fuller et al., 2006. Data collection methods were validated in previous studies of youth sports injury surveillance. | NR (no kappa/ICC/test–retest statistics reported). | Specific version of injury reporting forms not provided. |
| (Owoey  2014) | Overall injury, lower extremity injury, non-contact injury, injury by exposure type (match/training). | Time-loss injuries: injuries that caused players to be unable to fully participate in the next training session or match. | Injury classification based on location (lower extremity), mechanism (contact/non-contact), and severity (mild, moderate, severe). | Injuries were recorded weekly using injury report forms (IRFs), with interviews conducted to assess the mechanism, location, and severity. Coaches provided exposure data. | Injuries assessed by physiotherapists; follow-up through interviews by research assistants. | Weekly injury assessments during a 6-month season; players with injuries were assessed about a week after the incident. | Based on (Fuller et al. 2006)consensus guidelines for injury surveillance in football. | NR (no kappa/ICC/test–retest statistics reported). | NR |
| (Rössle  2018) | Overall injury, severe injury, lower extremity injury (knee, ankle, groin), traumatic fractures, overuse injuries, muscle and ligament injuries. | Any injury resulting in medical attention, removal from session, and/or absence from future training or match. | Injury classification based on location (lower extremities), type (muscle/ligament/traumatic fractures), and mechanism (contact/non-contact/overuse). | Online injury recording system; injury data were entered by coaches and monitored by study assistants. Players with injuries were interviewed and medical diagnoses were verified when necessary. | Injuries assessed by study assistants and physiotherapists. Medical diagnoses verified by treating physicians. | Weekly injury reporting during the football season (2014–2015). Follow-up for injuries conducted throughout the season. | Based on established football injury definitions (Fuller et al., 2006) and previously validated injury surveillance protocols. | NR (no kappa/ICC/test–retest statistics reported).. | NR |
| (Soligar  2008) | Overall injury, lower extremity injury (knee, ankle, lower leg, hip), acute injury, overuse injury. | Reportable injury: occurred during scheduled match or training, causing player to be unable to fully participate in the next session. | Injury classified by location (lower extremity), mechanism (contact/non-contact), and severity (minimal, mild, moderate, severe). | Weekly injury and exposure data recorded by coaches on registration forms; injuries recorded by physiotherapists. Follow-up by phone interviews with injured players using a standardized questionnaire. | Injuries assessed by physiotherapists and coaches; diagnosis confirmed by physicians when necessary. | Weekly during the study period (March to October 2007). Follow-up conducted within 4 weeks after injury. | Based on consensus statement by Fuller et al. (2006). | NR (no kappa/ICC/test–retest statistics reported). | Specific details on injury recording tools not provided. |
| (Walden  2012) | Overall knee injuries, anterior cruciate ligament (ACL) injuries, severe knee injuries (absence >4 weeks), acute knee injuries. | Acute knee injury: occurred during training or match, with sudden onset, and led to inability to participate fully in future training or match play. ACL injuries included both first and recurrent injuries. | Full injury definition based on Fuller et al. (2006); ACL injuries classified by type (partial/total rupture), with associated knee joint injuries classified by medical professionals. | Injury data were recorded by study therapists using injury report forms and attendance sheets. Coaches reported exposure data; study physicians diagnosed and verified ACL injuries. | Injury assessments performed by study therapists; ACL diagnoses confirmed by study physicians. Magnetic resonance imaging (MRI) was used for ACL injuries. | Weekly injury surveillance during the 2009 football season; follow-up for injuries conducted within 1 week, particularly for ACL injuries. | Based on consensus injury definitions (Fuller et al., 2006). MRI and clinical examination used for ACL injuries. | NR (no kappa/ICC/test–retest statistics reported). | No specific details on injury recording forms or MRI usage protocol provided. |
| (Zarei 2019) | Overall injury incidence, lower extremity injuries (knee, ankle, groin), time-loss injuries, match and training injuries. | Any injury leading to absence from the subsequent training or match. | Injury classified by body region (lower extremities), mechanism (contact/non-contact), and severity (time-loss injury classification). | Injury and exposure data were recorded using electronic forms by coaches. Injury characteristics were gathered using a standardized form from players and parents via phone/e-mail. | Injuries recorded by club physiotherapists or physicians. Follow-up by study assistants. | Injury and exposure data were recorded weekly throughout the football season (Feb–Nov 2017). Follow-up was done immediately after injury, with contact made with players, coaches, and parents. | Based on the FIFA 11+ Kids program injury prevention guidelines. | NR (no kappa/ICC/test–retest statistics reported). | No additional technical details on tools provided. |

**Supplementary Table S4A. Injury definition and assessment methods**

**Supplementary Table S4B. Instrument/device technical specifications**

| **Study (Author, Year)** | **Instrument / tool / form (name)** | **Sampling frequency (if applicable)** | **Manufacturer / brand** | **Model / version** | **Country of manufacture** | **Validity / validation ref(s) (of tool/device)** | **Notes (NR/unclear)** |
| --- | --- | --- | --- | --- | --- | --- | --- |
| (Steffen 2008) | Web-based injury & exposure recording system; standardized injury questionnaire (name/version NR); exposure/compliance recording forms. | Continuous recording; coaches contacted at least monthly (telephone/e-mail) for exposure and injury updates. | NR | NR | NR | Fuller et al., 2006; van Mechelen et al., 1992; method reliability/validity discussed previously (Olsen et al., 2006). | NR |
| (Al Attar 2023) | Sports Injury Tracker (Australian Sports Injury Data Dictionary form). | Weekly injury data collection. | Australian Sports Injury Data Dictionary. | Version for soccer injury tracking. | Australia. | Based on the Australian Sports Injury Data Dictionary. | NR |
| (Emery 2010) | Injury report forms (IRFs) and weekly exposure sheets (WES).(Emery et al. 2005) | Weekly injury data and exposure data collection during the season.. | NR | Version of IRF used for injury surveillance in youth soccer. | NR | Based on previous work validating the injury report forms in adolescent soccer populations . | NR |
| (Hilska 2021) | SMS injury reporting system; structured injury questionnaire for follow-up interviews. | Weekly injury data collection via SMS; interviews conducted after each injury report. | NR | NR | NR | Based on previously validated injury reporting methods in youth soccer populations (Fuller et al., 2006). | NR |
| (Owoeye 2014) | Injury report forms (IRFs) and exposure forms. | Weekly injury and exposure data collection throughout the 6-month season. | NR | NR |  | Based on Fuller et al. (2006) injury surveillance guidelines. | NR |
| (Rössler 2018) | Online injury recording system (web-based platform), injury reporting forms. | Weekly injury data collection during the season. | NR | Web-based system adapted from previous injury surveillance systems. | NR | Based on injury surveillance methods validated in prior studies (Fuller et al., 2006). | NR |
| (Soligard 2008) | Injury report forms (IRFs), weekly exposure registration forms. | Weekly injury and exposure data collection during the study period. | NR | NR | NR | Based on established injury surveillance protocols, Fuller et al. (2006). | No specific details provided for the version or technical specifications of the injury reporting forms. |
| (Walden 2012) | Injury report forms (IRFs) and weekly attendance sheets. | Weekly injury and exposure data collection during the season. | NR | NR | NR | Based on Fuller et al. (2006) injury surveillance protocols. | No additional details on technical specifications provided. |
| (Zarei 2019) | Electronic exposure and injury reporting forms. | Weekly data collection on injuries and exposure during the season. | NR | NR | NR | Based on the FIFA 11+ Kids program guidelines for injury reporting. | Specific tool/device version details not provided. |

*Abbreviations: NR = not reported ，NR indicates that the corresponding technical specifications were not reported in the original publications and could not be retrieved from supplementary materials.; ref = reference; AT = athletic trainer; ICC = intraclass correlation coefficient.*

Emery CA, Meeuwisse WH, and Hartmann SE. 2005. Evaluation of risk factors for injury in adolescent soccer: implementation and validation of an injury surveillance system. *Am J Sports Med* 33:1882-1891. 10.1177/0363546505279576

Fuller CW, Ekstrand J, Junge A, Andersen TE, Bahr R, Dvorak J, Hagglund M, McCrory P, and Meeuwisse WH. 2006. Consensus statement on injury definitions and data collection procedures in studies of football (soccer) injuries. *Br J Sports Med* 40:193-201. 10.1136/bjsm.2005.025270

Olsen OE, Myklebust G, Engebretsen L, and Bahr R. 2006. Injury pattern in youth team handball: a comparison of two prospective registration methods. *Scand J Med Sci Sports* 16:426-432. 10.1111/j.1600-0838.2005.00484.x

**Table S5. Inter-rater agreement for study selection and risk-of-bias assessment (Cohen’s kappa)**

| **Record_ID** | | **Reviewer_A** | | **Reviewer_B** | |  | | | **Summary** | |  | | |  |  |  |  |  |
| --- | --- | --- | --- | --- | --- | --- | --- | --- | --- | --- | --- | --- | --- | --- | --- | --- | --- | --- |
| Al Attar2023 | | Include | | Include | |  | | | a = both Include | | 9 | | |  |  |  |  |  |
| Emery2010 | | Include | | Include | |  | | | b = A Include / B Exclude | | 0 | | |  |  |  |  |  |
| Hilska2021 | | Include | | Include | |  | | | c = A Exclude / B Include | | 5 | | |  |  |  |  |  |
| Owoeye 2014 | | Include | | Include | |  | | | d = both Exclude | | 21 | | |  |  |  |  |  |
| Rossler2018 | | Include | | Include | |  | | | N | | 35 | | |  |  |  |  |  |
| soligard 2008 | | Include | | Include | |  | | | Observed agreement (Po) | | 0.857142857 | | |  |  |  |  |  |
| steffen 2008 | | Include | | Include | |  | | | Chance agreement (Pe) | | 0.548571429 | | |  |  |  |  |  |
| walden 2012 | | Include | | Include | |  | | | Cohen's kappa | | 0.683544304 | | |  |  |  |  |  |
| Zarei 2019 | | Include | | Include | |  | | |  | |  | | |  |  |  |  |  |
| Ashley 2017 | | Exclude | | Exclude | |  | | |  | |  | | |  |  |  |  |  |
| Brunelli 2025 | | Exclude | | Exclude | |  | | |  | |  | | |  |  |  |  |  |
| Althomali 2025 | | Exclude | | Exclude | |  | | |  | |  | | |  |  |  |  |  |
| Wang 2024 | | Exclude | | Exclude | |  | | |  | |  | | |  |  |  |  |  |
| Lindblom 2023 | | Exclude | | Exclude | |  | | |  | |  | | |  |  |  |  |  |
| Patterson 2022 | | Exclude | | Exclude | |  | | |  | |  | | |  |  |  |  |  |
| Veith 2021 | | Exclude | | Exclude | |  | | |  | |  | | |  |  |  |  |  |
| Rahlf 2020 | | Exclude | | Exclude | |  | | |  | |  | | |  |  |  |  |  |
| Hilska 2018 | | Exclude | | Include | |  | | |  | |  | | |  |  |  |  |  |
| Thompson 2017 | | Exclude | | Exclude | |  | | |  | |  | | |  |  |  |  |  |
| O'Brien 2017 | | Exclude | | Exclude | |  | | |  | |  | | |  |  |  |  |  |
| Faude 2017 | | Exclude | | Exclude | |  | | |  | |  | | |  |  |  |  |  |
| Zebis 2016 | | Include | | Enclude | |  | | |  | |  | | |  |  |  |  |  |
| Wingfield 2013 | | Exclude | | Include | |  | | |  | |  | | |  |  |  |  |  |
| Al Attar 2016 | | Exclude | | Include | |  | | |  | |  | | |  |  |  |  |  |
| van 2012 | | Exclude | | Exclude | |  | | |  | |  | | |  |  |  |  |  |
| Frew2012 | | Exclude | | Exclude | |  | | |  | |  | | |  |  |  |  |  |
| Kiani 2010 | | Exclude | | Exclude | |  | | |  | |  | | |  |  |  |  |  |
| Zouita 2016 | | Exclude | | Include | |  | | |  | |  | | |  |  |  |  |  |
| Meha 2025 | | Exclude | | Exclude | |  | | |  | |  | | |  |  |  |  |  |
| García 2025 | | Exclude | | Exclude | |  | | |  | |  | | |  |  |  |  |  |
| Zhang 2024 | | Exclude | | Exclude | |  | | |  | |  | | |  |  |  |  |  |
| Valentin 2024 | | Exclude | | Include | |  | | |  | |  | | |  |  |  |  |  |
| Saber 2024 | | Exclude | | Exclude | |  | | |  | |  | | |  |  |  |  |  |
| Iatropoulos 2024 | | Exclude | | Exclude | |  | | |  | |  | | |  |  |  |  |  |
| Torres 2023 | | Exclude | | Exclude | |  | | |  | |  | | |  |  |  |  |  |
| Cotellessa 2023 | | Exclude | | Exclude | |  | | |  | |  | | |  |  |  |  |  |
| **Risk-of-bias kappa ( categories: Low / Unclear / High)** | | | | | | | | | | | | | | | | | | |
|  |  | |  | |  | |  |  | |  | |  |  | | | | | |
| **Study** | **Domain** | | **Reviewer A** | | **Reviewer B** | | **Confusionmatrix** | **B: Low** | | **B: Unclear** | | **B:High** | **Row total** | | | | | |
| Al Attar2023 | Random sequence generation | | Low | | Low | | A: Low | 38 | | 4 | | 0 | 42 | | | | | |
| Al Attar2023 | Allocation concealment | | Low | | Low | | A: Unclear | 1 | | 9 | | 0 | 10 | | | | | |
| Al Attar2023 | Blinding of outcome assessment | | Low | | Unclear | | A: High | 0 | | 0 | | 2 | 2 | | | | | |
| Al Attar2023 | Selective outcome reporting | | Low | | Low | | Column total |  | |  | |  |  | | | | | |
| Al Attar2023 | Other risks of bias | | Low | | Low | |  | 39 | | 13 | | 2 | 54 | | | | | |
| Al Attar2023 | Incomplete outcome data | | Unclear | | Low | |  |  | |  | |  |  | | | | | |
|  |  | |  | |  | | Summary |  | |  | |  |  | | | | | |
|  |  | |  | |  | | Observed agreement (Po) | 0.907407407 | |  | |  |  | | | | | |
|  |  | |  | |  | | Chance agreement (Pe) | 0.607681756 | |  | |  |  | | | | | |
|  |  | |  | |  | | Cohen's kappa | 0.763986014 | |  | |  |  | | | | | |
| Emery2010 | Random sequence generation | | Low | | Low | | Interpretation | Substantial | |  | |  |  | | | | | |
| Emery2010 | Allocation concealment | | Low | | Low | |  |  | |  | |  |  | | | | | |
| Emery2010 | Blinding of outcome assessment | | Low | | Unclear | | Note | Use only Low / Unclear / High | |  | |  |  | | | | | |
| Emery2010 | Selective outcome reporting | | Low | | Low | |  |  | |  | |  |  | | | | | |
| Emery2010 | Other risks of bias | | High | | High | |  |  | |  | |  |  | | | | | |
| Emery2010 | Incomplete outcome data | | Unclear | | Unclear | |  |  | |  | |  |  | | | | | |
|  |  | |  | |  | |  |  | |  | |  |  | | | | | |
| Hilska2021 | Random sequence generation | | Low | | Low | |  |  | |  | |  |  | | | | | |
| Hilska2021 | Allocation concealment | | Low | | Low | |  |  | |  | |  |  | | | | | |
| Hilska2021 | Blinding of outcome assessment | | Low | | low | |  |  | |  | |  |  | | | | | |
| Hilska2021 | Selective outcome reporting | | Low | | Low | |  |  | |  | |  |  | | | | | |
| Hilska2021 | Other risks of bias | | Low | | Low | |  |  | |  | |  |  | | | | | |
| Hilska2021 | Incomplete outcome data | | Unclear | | Unclear | |  |  | |  | |  |  | | | | | |
|  |  | |  | |  | |  |  | |  | |  |  | | | | | |
| Owoeye 2014 | Random sequence generation | | Low | | Low | |  |  | |  | |  |  | | | | | |
| Owoeye 2014 | Allocation concealment | | Low | | Low | |  |  | |  | |  |  | | | | | |
| Owoeye 2014 | Blinding of outcome assessment | | High | | High | |  |  | |  | |  |  | | | | | |
| Owoeye 2014 | Selective outcome reporting | | Low | | Low | |  |  | |  | |  |  | | | | | |
| Owoeye 2014 | Other risks of bias | | Low | | Low | |  |  | |  | |  |  | | | | | |
| Owoeye 2014 | Incomplete outcome data | | Unclear | | Unclear | |  |  | |  | |  |  | | | | | |
|  |  | |  | |  | |  |  | |  | |  |  | | | | | |
| Rossler2018 | Random sequence generation | | Low | | Low | |  |  | |  | |  |  | | | | | |
| Rossler2018 | Allocation concealment | | Low | | Low | |  |  | |  | |  |  | | | | | |
| Rossler2018 | Blinding of outcome assessment | | Low | | Unclear | |  |  | |  | |  |  | | | | | |
| Rossler2018 | Selective outcome reporting | | Low | | Low | |  |  | |  | |  |  | | | | | |
| Rossler2018 | Other risks of bias | | Low | | Low | |  |  | |  | |  |  | | | | | |
| Rossler2018 | Incomplete outcome data | | Unclear | | Unclear | |  |  | |  | |  |  | | | | | |
|  |  | |  | |  | |  |  | |  | |  |  | | | | | |
| soligard 2008 | Random sequence generation | | Low | | Low | |  |  | |  | |  |  | | | | | |
| soligard 2008 | Allocation concealment | | Low | | Low | |  |  | |  | |  |  | | | | | |
| soligard 2008 | Blinding of outcome assessment | | Low | | Low | |  |  | |  | |  |  | | | | | |
| soligard 2008 | Selective outcome reporting | | Low | | Low | |  |  | |  | |  |  | | | | | |
| soligard 2008 | Other risks of bias | | Low | | Low | |  |  | |  | |  |  | | | | | |
| soligard 2008 | Incomplete outcome data | | Unclear | | Unclear | |  |  | |  | |  |  | | | | | |
|  |  | |  | |  | |  |  | |  | |  |  | | | | | |
| steffen 2008 | Random sequence generation | | Low | | Low | |  |  | |  | |  |  | | | | | |
| steffen 2008 | Allocation concealment | | Low | | Low | |  |  | |  | |  |  | | | | | |
| steffen 2008 | Blinding of outcome assessment | | Low | | Unclear | |  |  | |  | |  |  | | | | | |
| steffen 2008 | Selective outcome reporting | | Low | | Low | |  |  | |  | |  |  | | | | | |
| steffen 2008 | Other risks of bias | | Low | | Low | |  |  | |  | |  |  | | | | | |
| steffen 2008 | Incomplete outcome data | | Unclear | | Unclear | |  |  | |  | |  |  | | | | | |
|  |  | |  | |  | |  |  | |  | |  |  | | | | | |
| walden 2012 | Random sequence generation | | Low | | Low | |  |  | |  | |  |  | | | | | |
| walden 2012 | Allocation concealment | | Low | | Low | |  |  | |  | |  |  | | | | | |
| walden 2012 | Blinding of outcome assessment | | Unclear | | Unclear | |  |  | |  | |  |  | | | | | |
| walden 2012 | Selective outcome reporting | | Low | | Low | |  |  | |  | |  |  | | | | | |
| walden 2012 | Other risks of bias | | Low | | Low | |  |  | |  | |  |  | | | | | |
| walden 2012 | Incomplete outcome data | | Unclear | | Unclear | |  |  | |  | |  |  | | | | | |
|  |  | |  | |  | |  |  | |  | |  |  | | | | | |
| Zarei 2019 | Random sequence generation | | Low | | Low | |  |  | |  | |  |  | | | | | |
| Zarei 2019 | Allocation concealment | | Low | | Low | |  |  | |  | |  |  | | | | | |
| Zarei 2019 | Blinding of outcome assessment | | Low | | Low | |  |  | |  | |  |  | | | | | |
| Zarei 2019 | Selective outcome reporting | | Low | | Low | |  |  | |  | |  |  | | | | | |
| Zarei 2019 | Other risks of bias | | Low | | Low | |  |  | |  | |  |  | | | | | |
| Zarei 2019 | Incomplete outcome data | | Unclear | | Unclear | |  |  | |  | |  |  | | | | | |


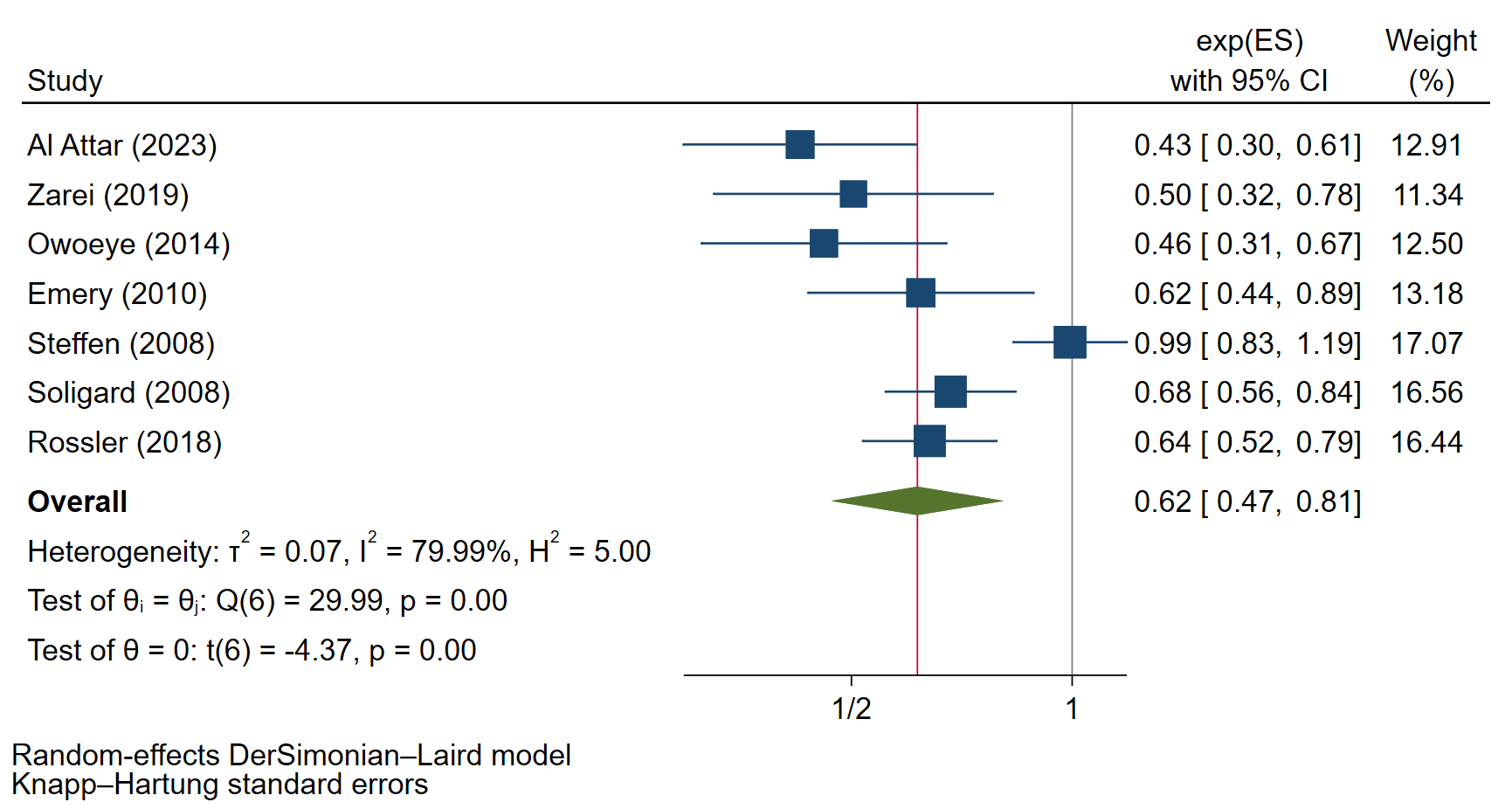


FigS1


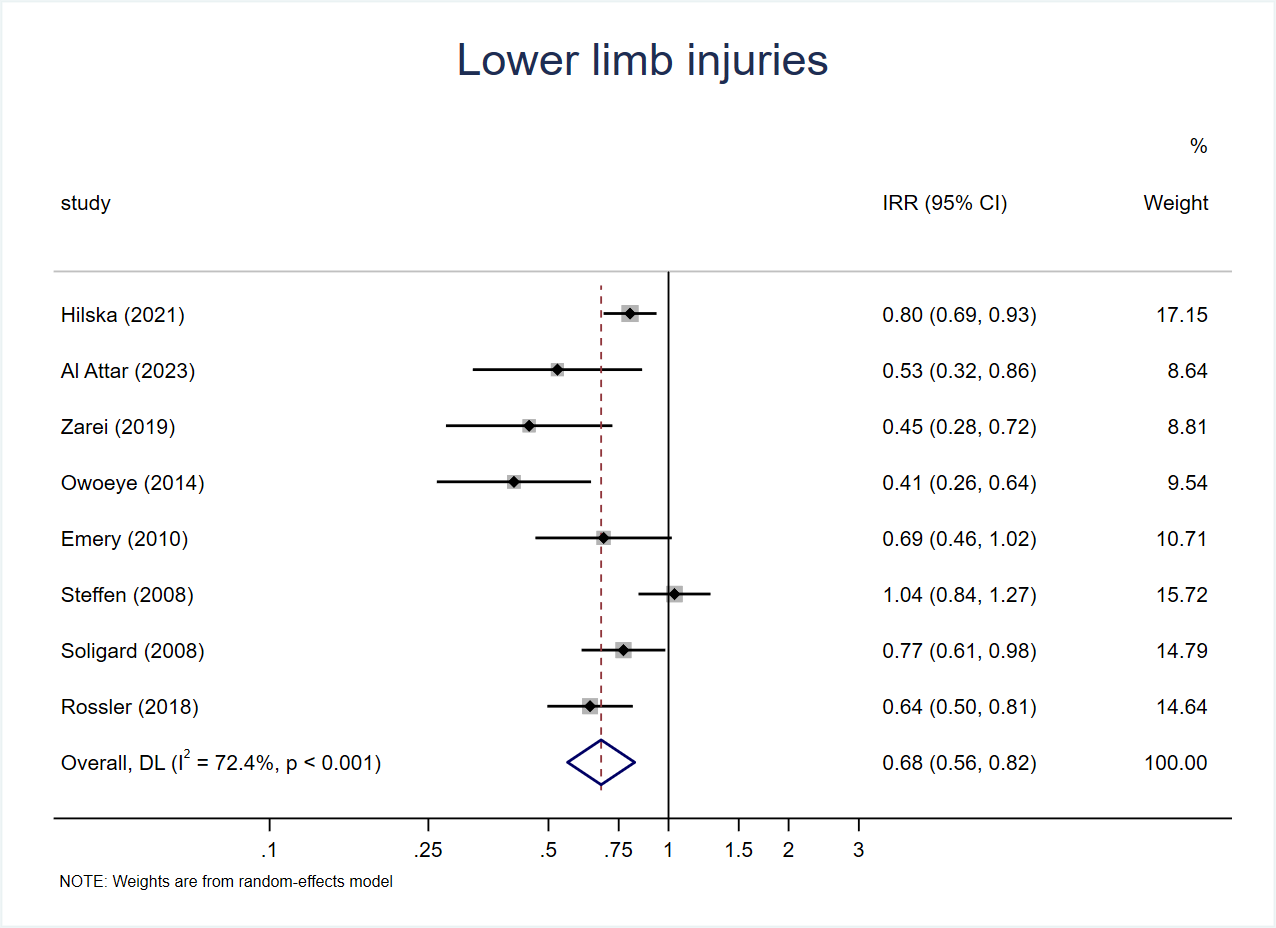


FigS2


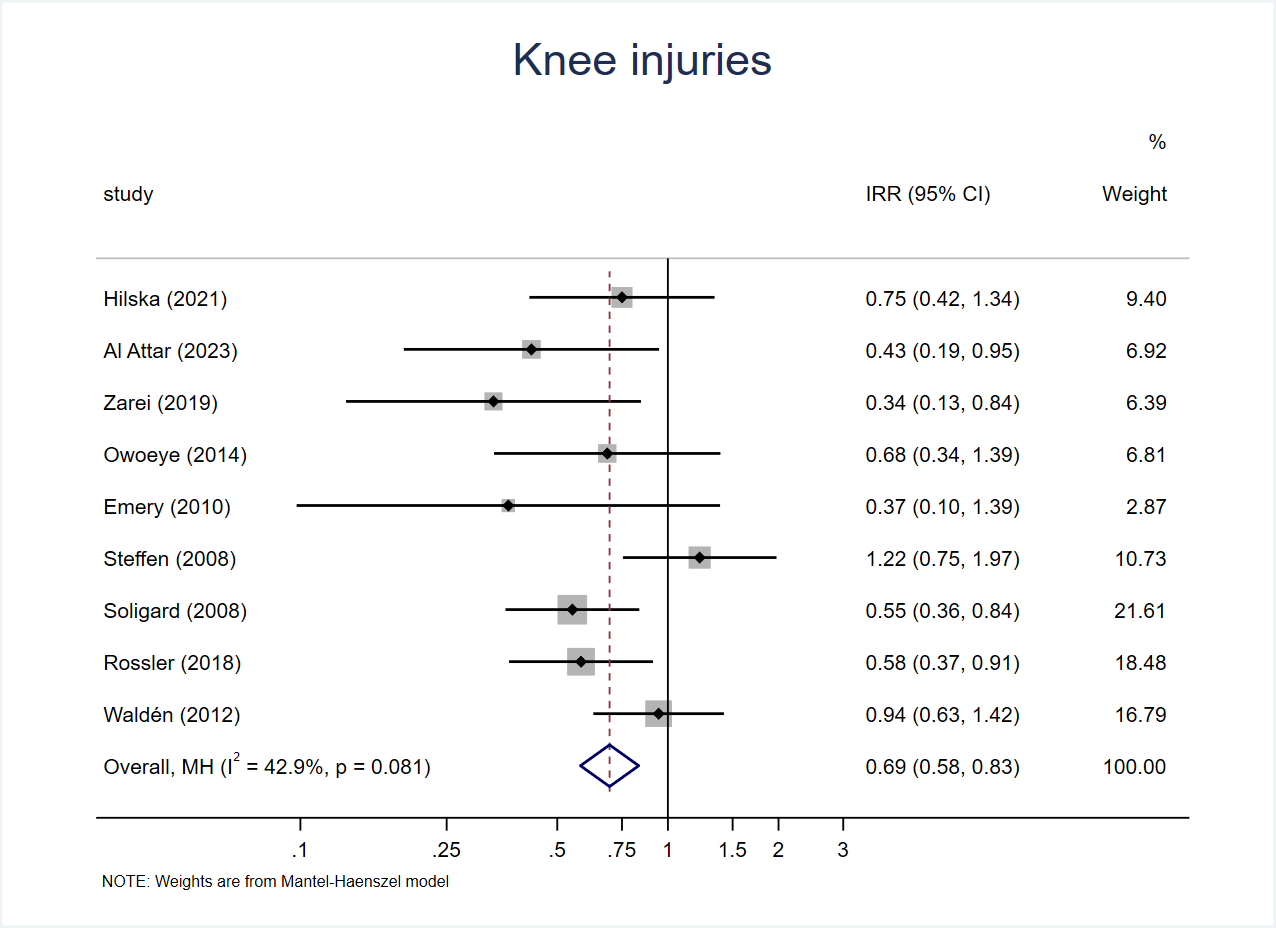


FigS3


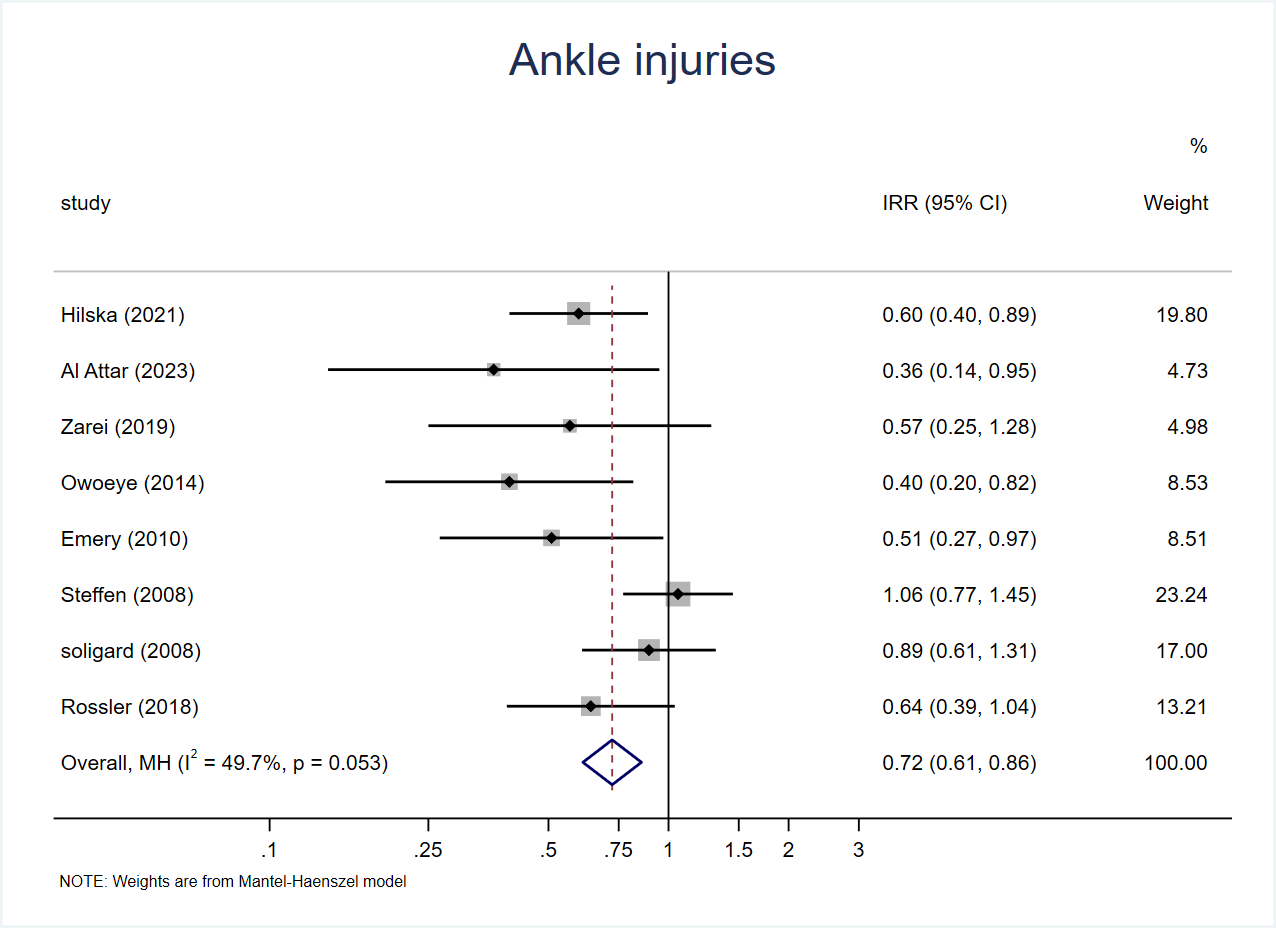


FigS4


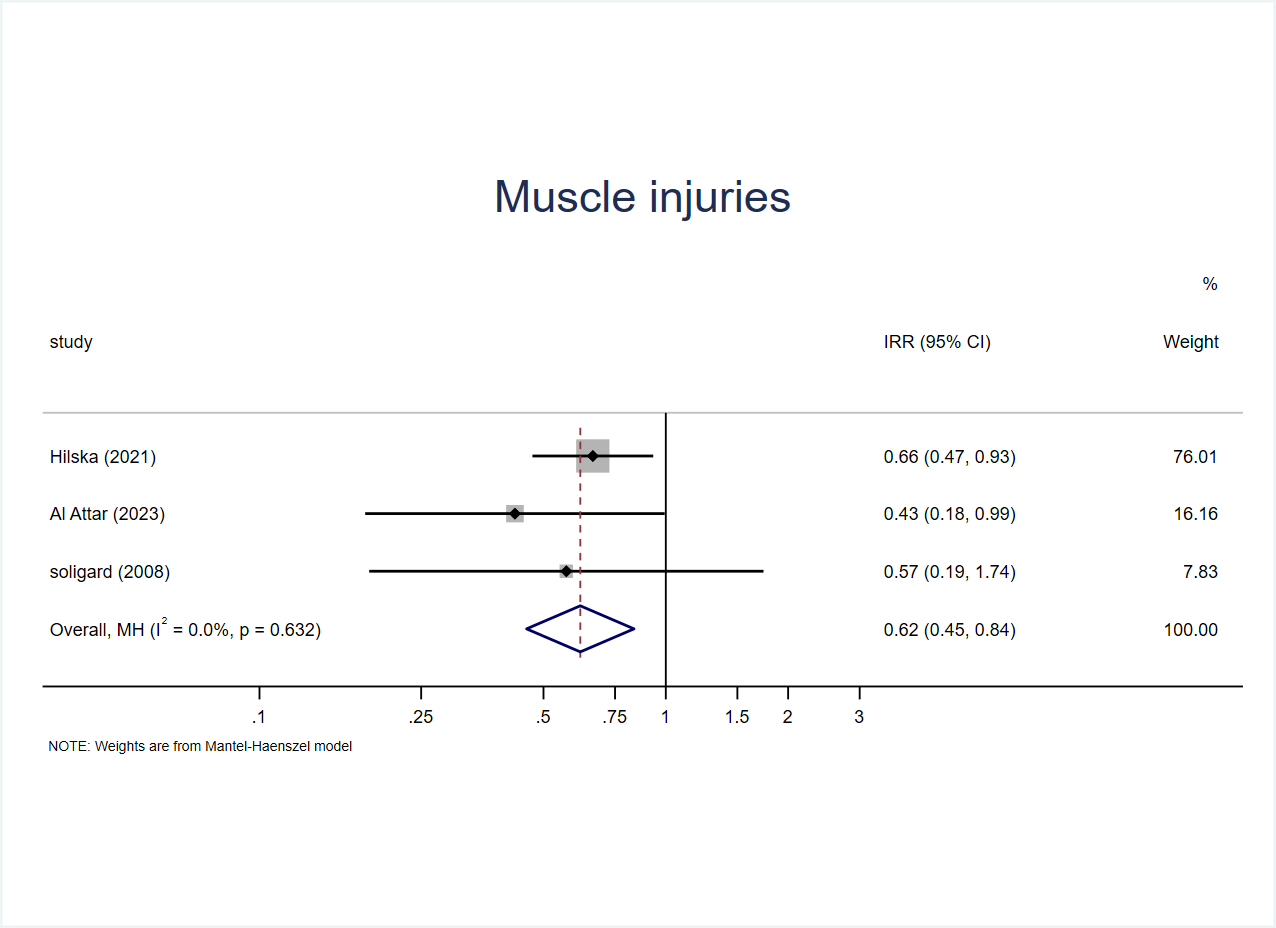


FigS5


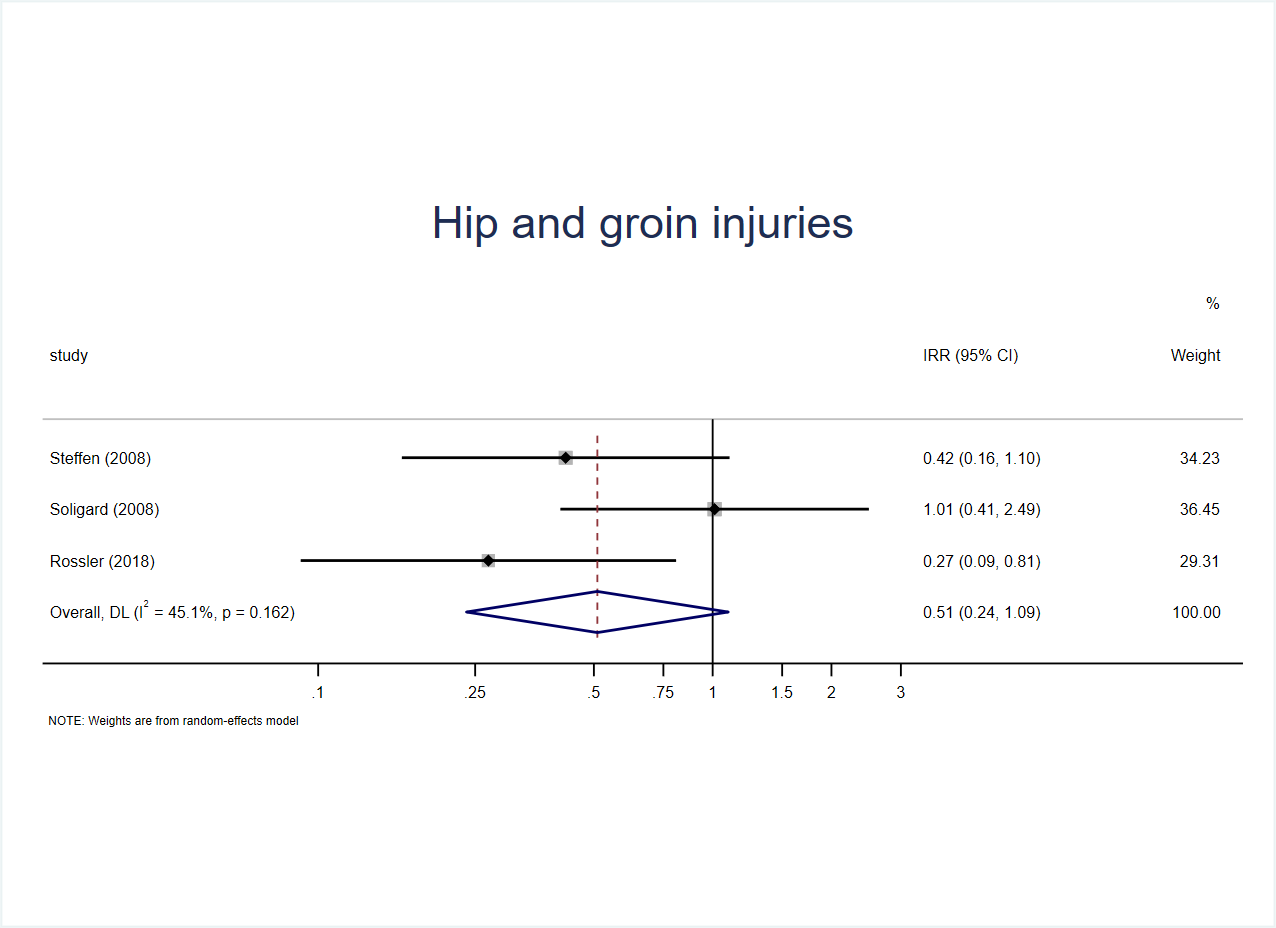


FigS6


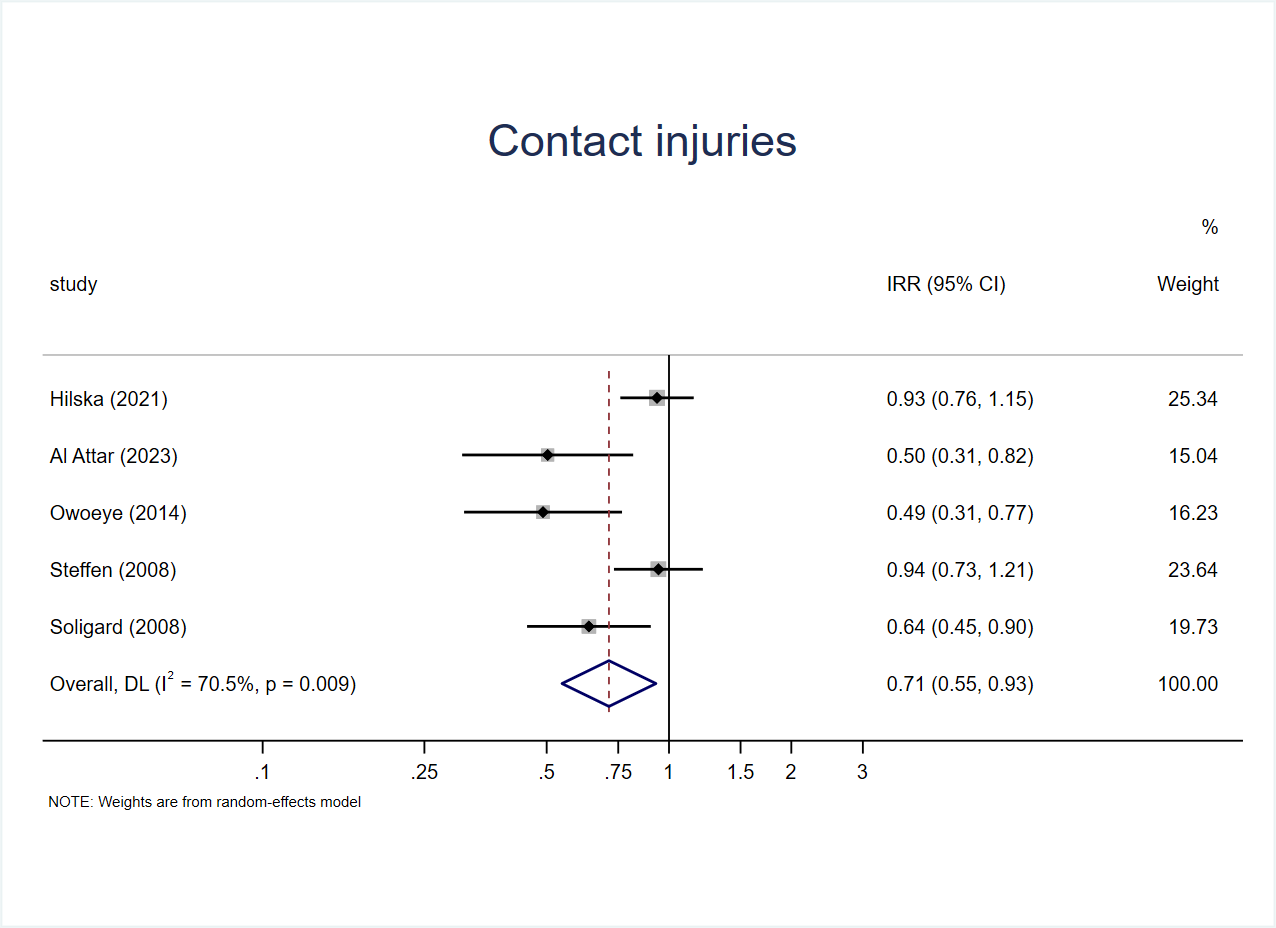


FigS7


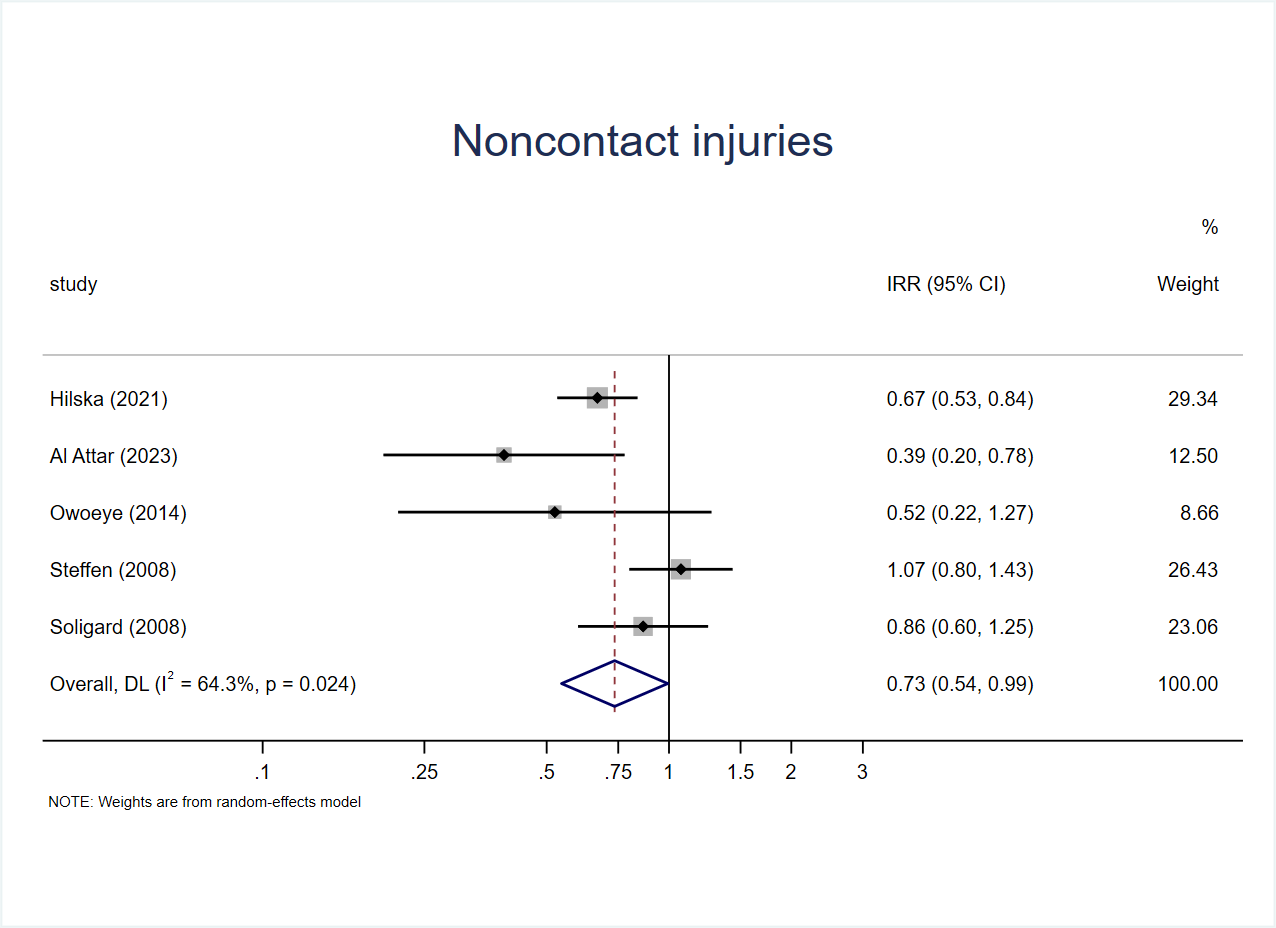


FigS8


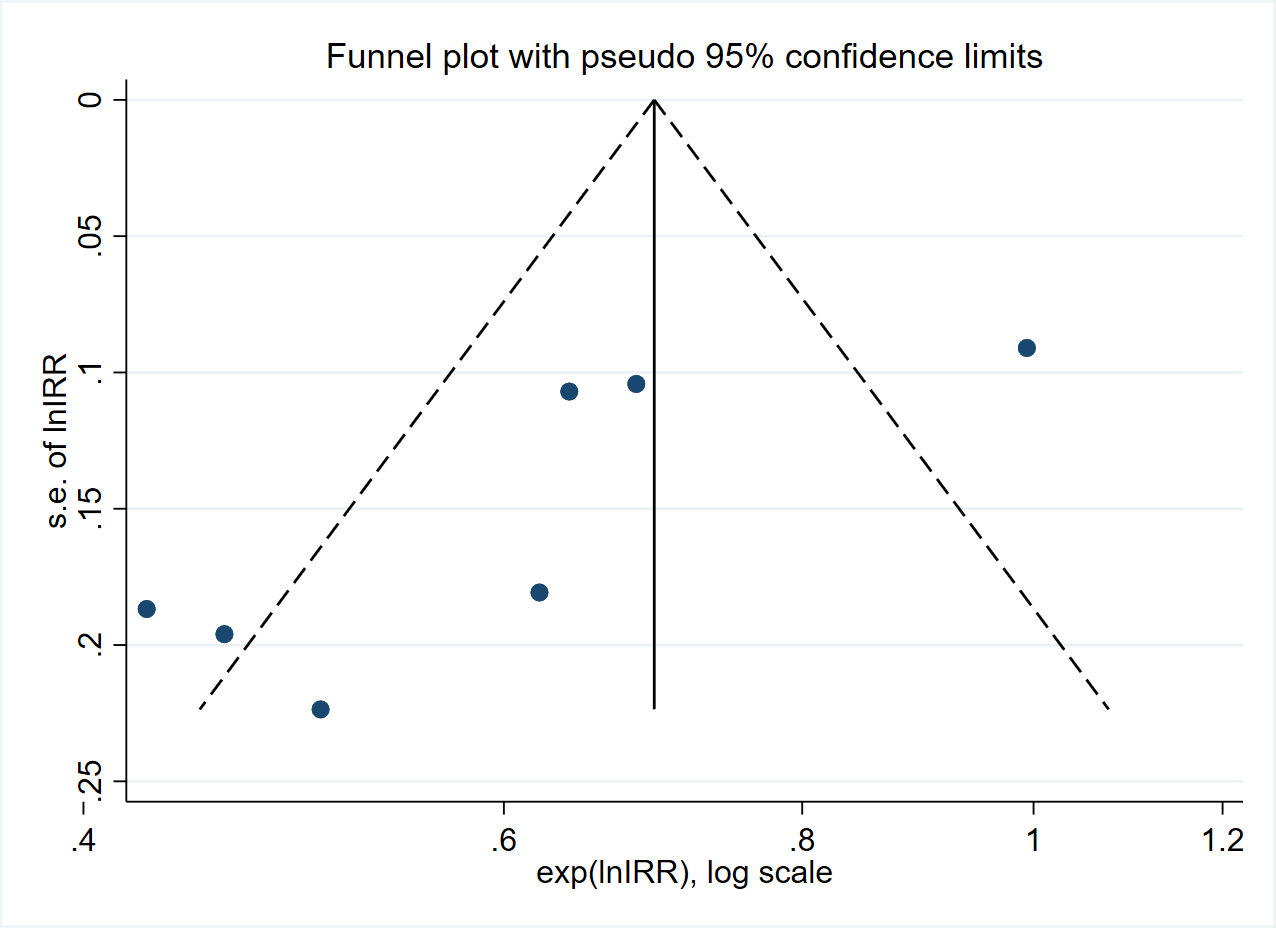


FigS9


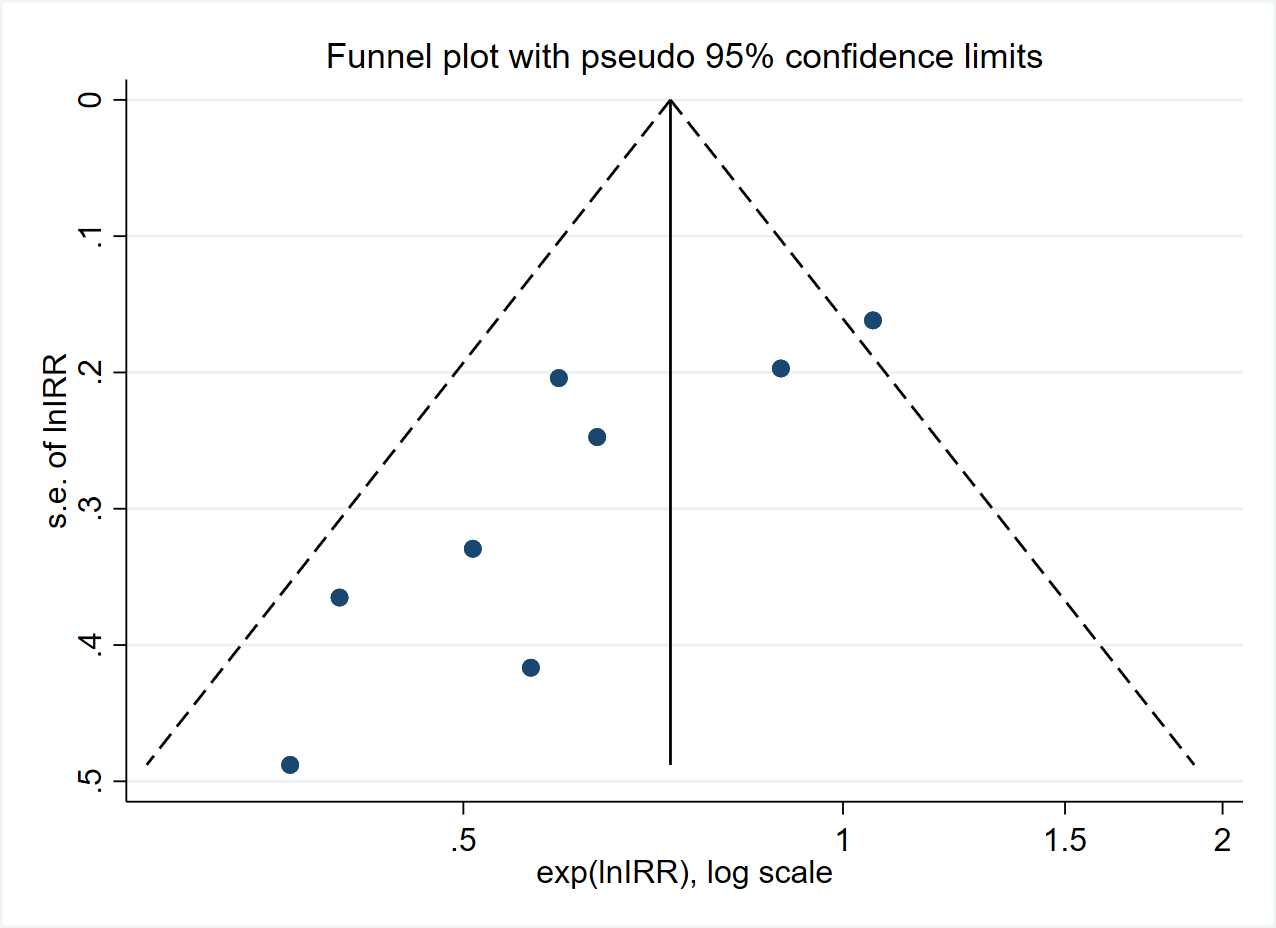


Fig S10


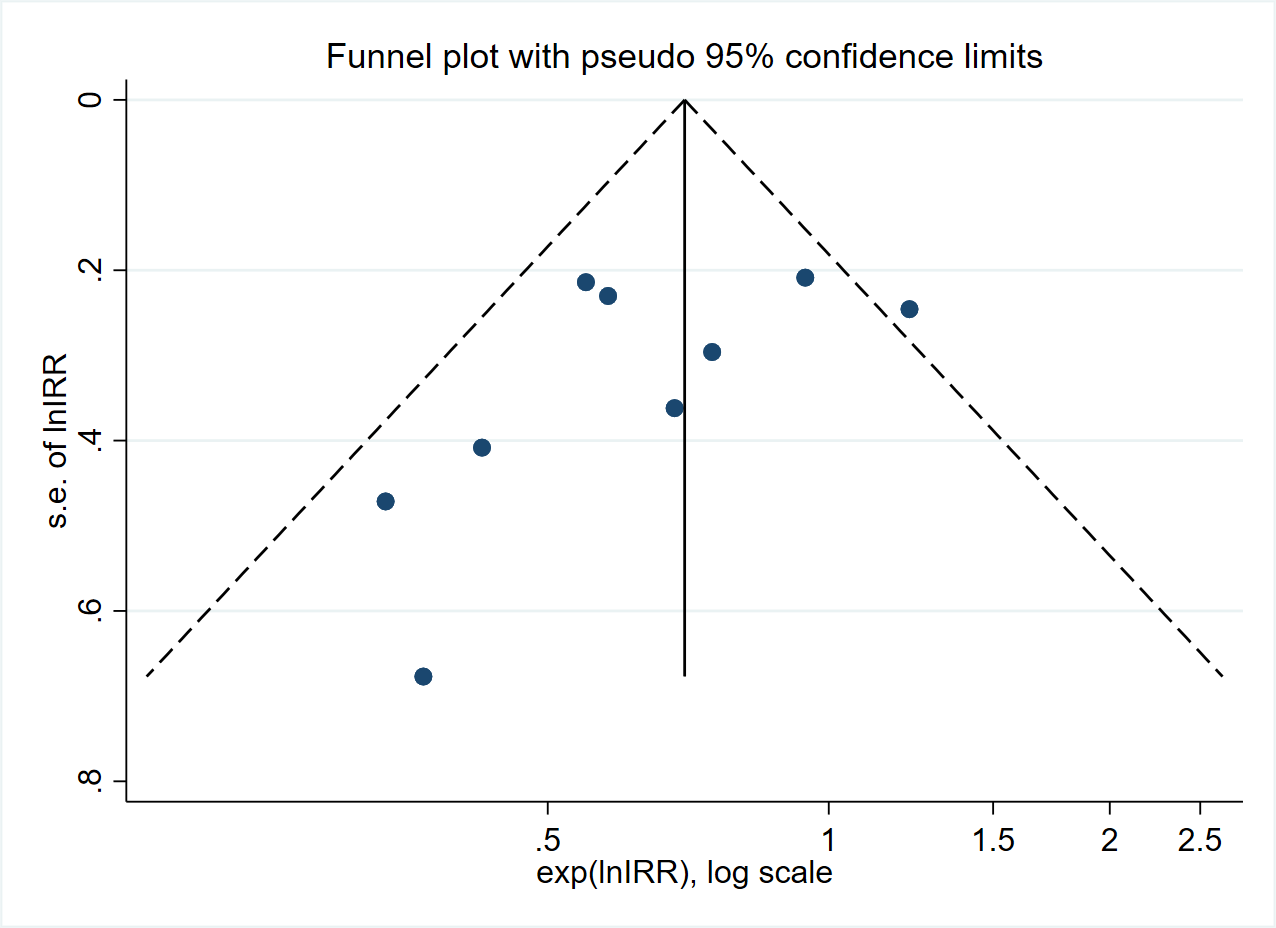


Fig S11


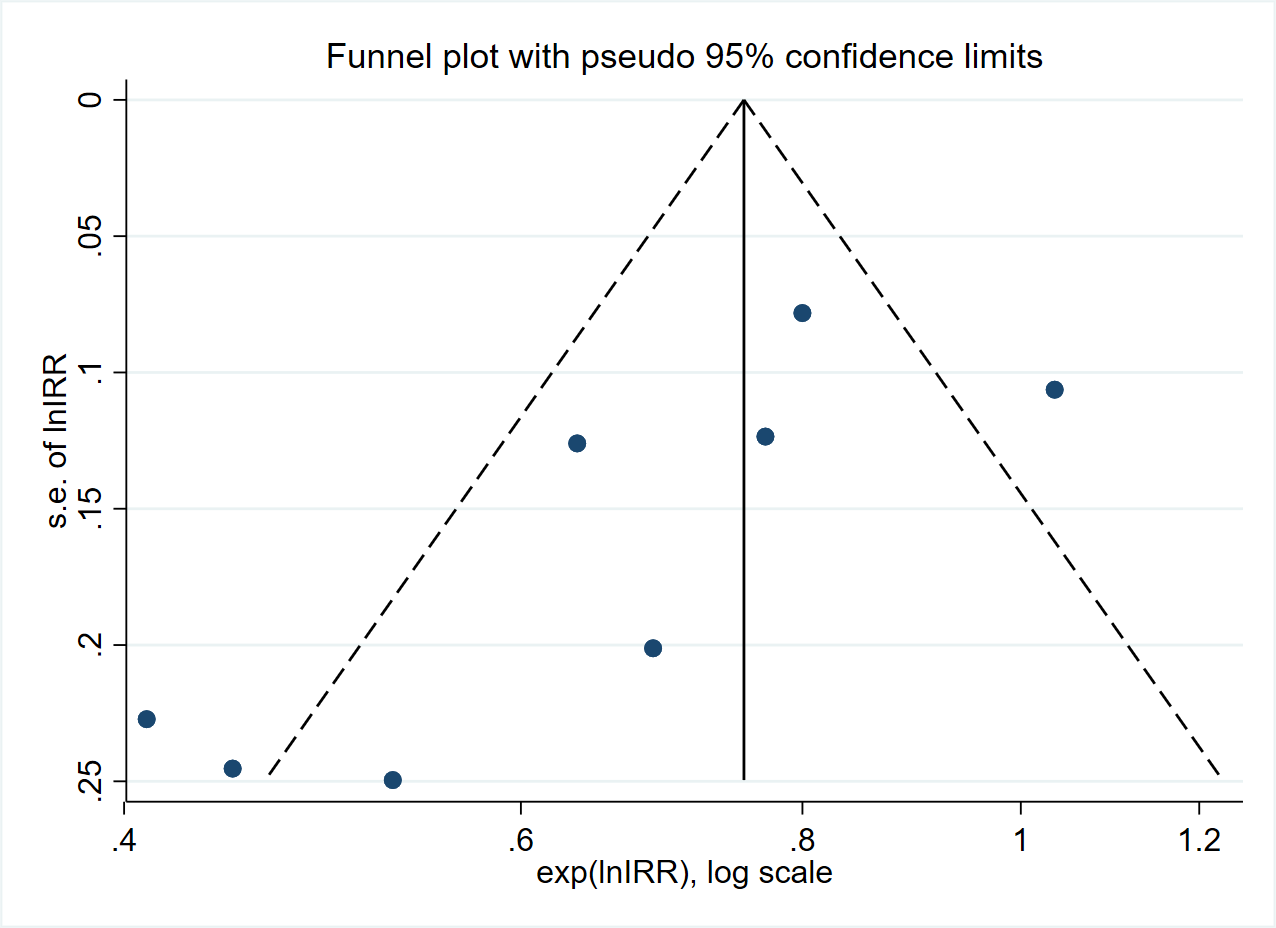


Fig S12


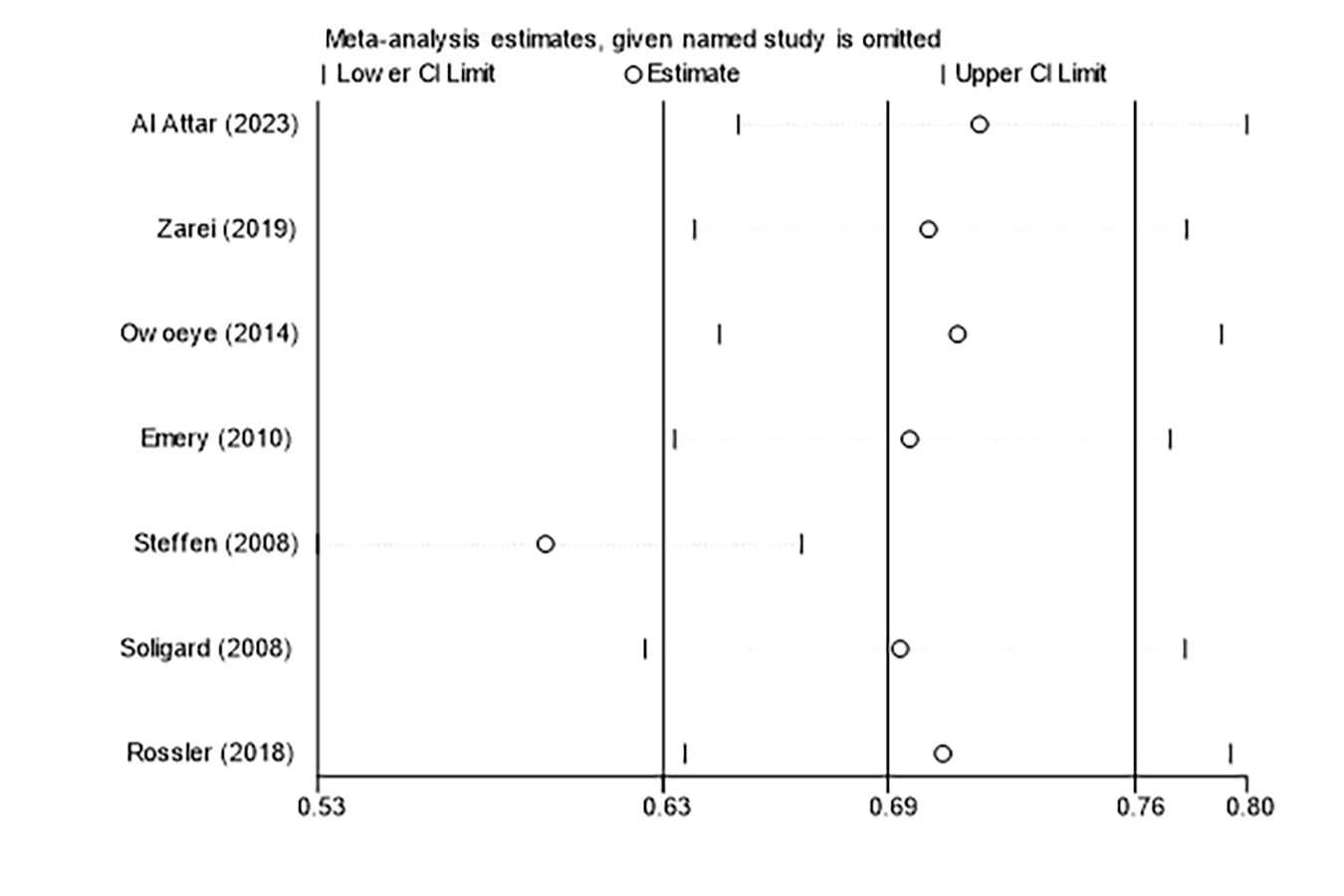


Fig S13


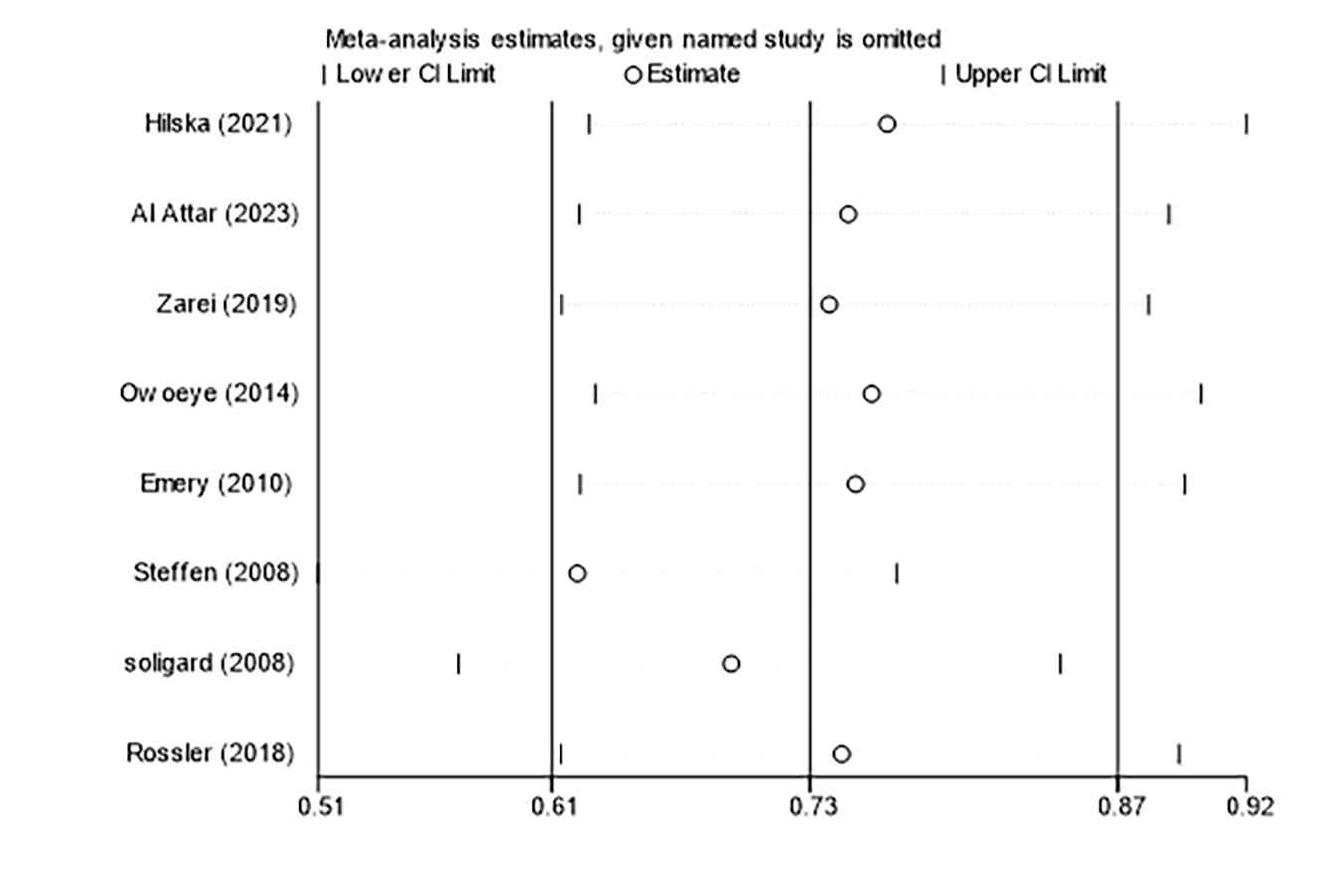


Fig S14


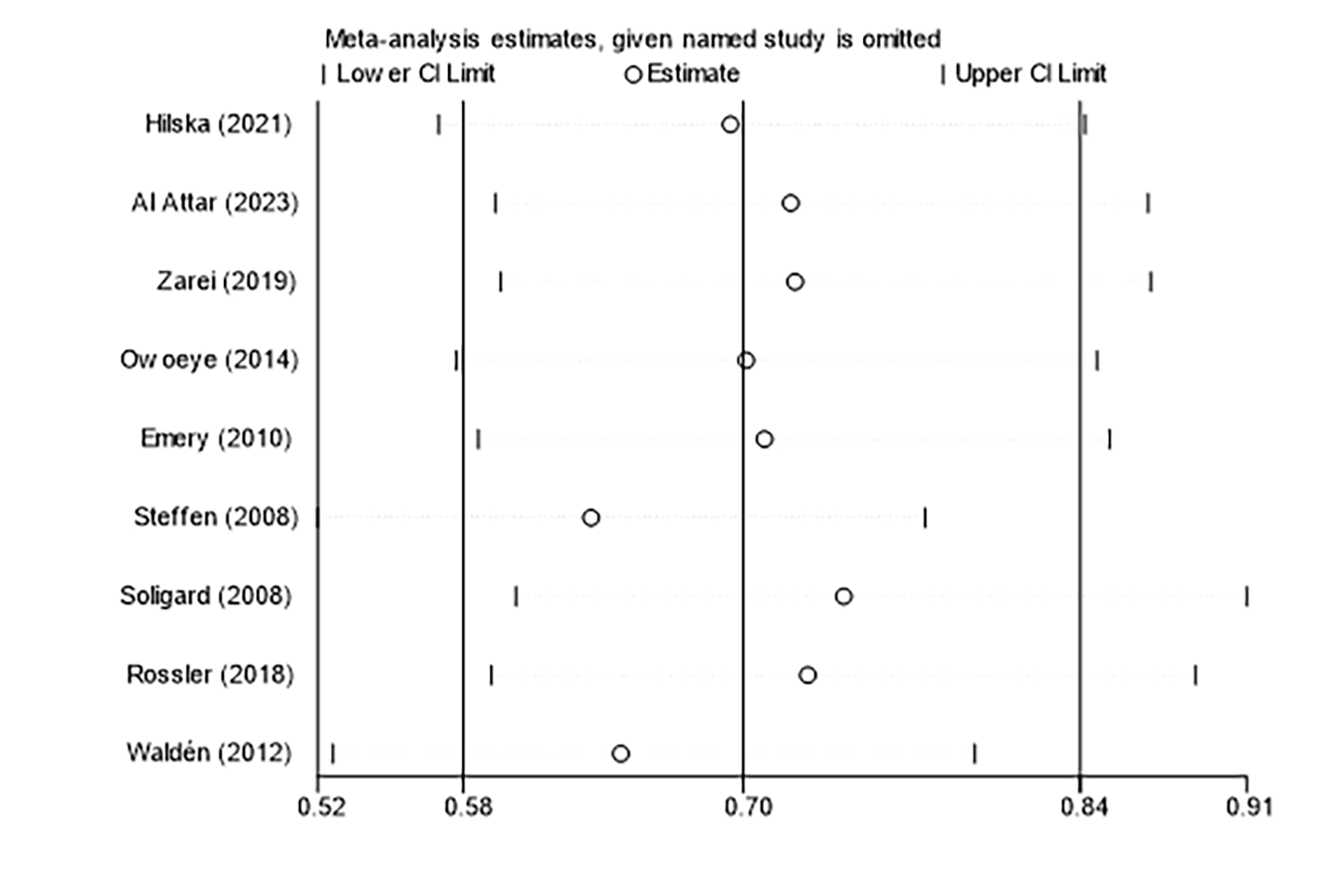


FigS15


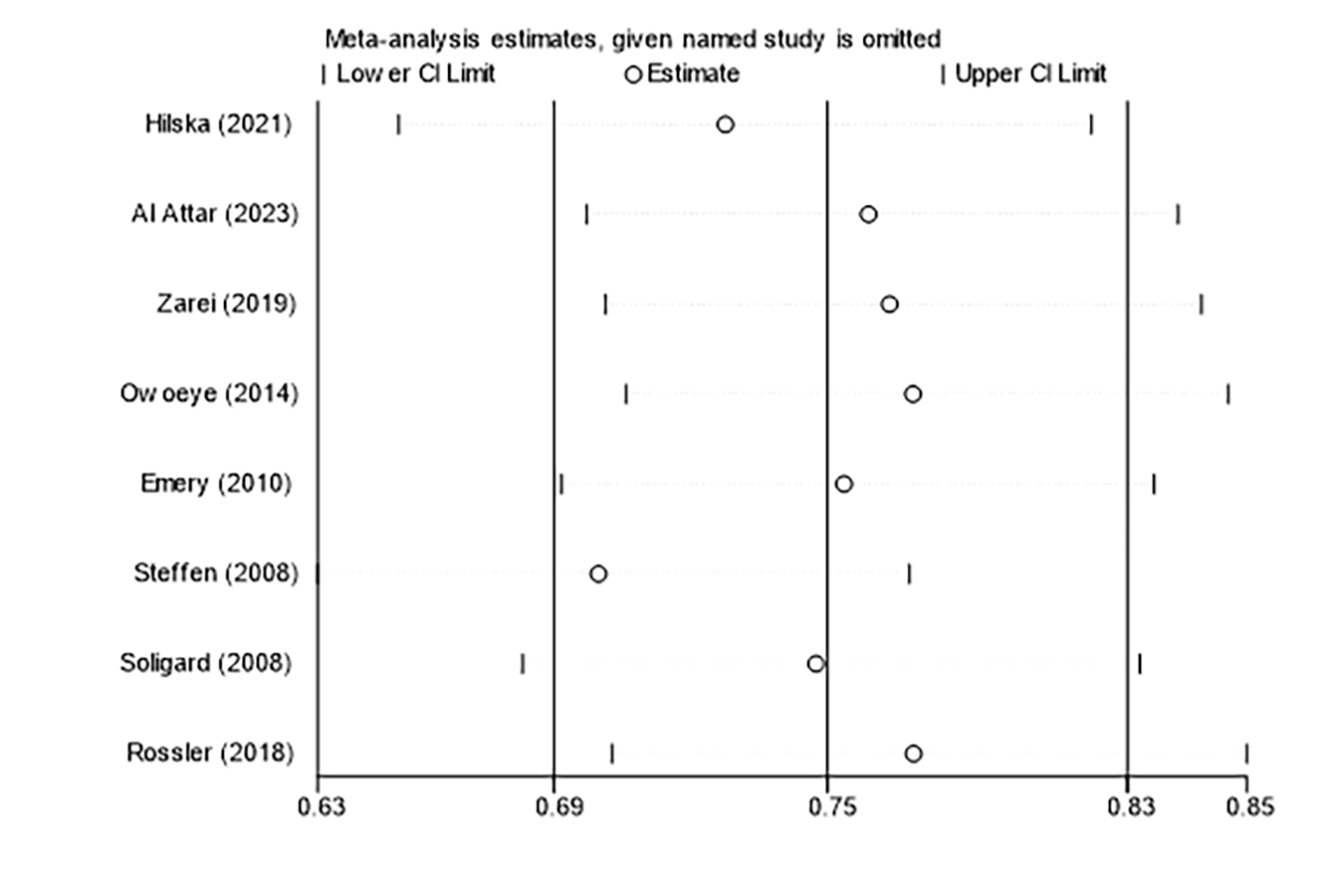


FigS16
